# Supplementary material for: Heuristics for the sustainable harvest of wildlife in stochastic social-ecological systems
Source: PLoS One. 2021 Nov 19;16(11):e0260159. doi: 10.1371/journal.pone.0260159 (PMC8604319; doi:10.1371/journal.pone.0260159)
Supplement: S1 Appendix — (DOCX) [file pone.0260159.s001.docx]

# Supporting information S1: MSE Model details

Accompanying manuscript: Heuristics for the sustainable harvest of wildlife in stochastic social-ecological systems.

Authors: Elizabeth Law, John D. C. Linnell, Bram van Moorter, Erlend B. Nilsen.

This supporting information repeats the methods presented in the main text, with additional detail where required, particularly regarding the assumptions and caveats.

## S1.1 Model framework

We develop an MSE model that generalises a terrestrial wildlife harvest system, with components of 1) resource dynamics, 2) monitoring observations, 3) quota setting, 4) harvest implementation, and 5) sustainability evaluation. Simulations occur in discrete yearly time steps (*t*), across a time series of 20 years (broadly considered long term for applied management plans), with multiple stochastic replicates (*i* = 1000) per scenario. An overall perspective is provided in figure S1.1.

The **resource population component** simulates growth of a population *N_i,t_*, via a logistic growth function determined by the population intrinsic growth rate, *r_i,t_*, and the carrying capacity, *K*.

(1) $N_{i,t+1}=N_{i,t}+r_{i,t}N_{i, t}\frac{(K- N_{i,t})}{K}$ (rounded to nearest non-negative integer)

where:

(2) $r_{i,t} \approx N(r_{i}^{m}, r_{i}^{sd})$

(3) $r_{i}^{m} \approx N(r^{mm}, r^{sdm})$

(4) $r_{i}^{sd} \approx N(r^{msd}, r^{sdsd})$

We assume that $r^{mm}, r^{sdm}, r^{msd}, r^{sdsd}$, and *K* are constant for each species context and variation scenario. Parameters for these are given below. Thus, variation in *r* is simulated by a normal distribution, equally partitioned over years and replicates. Yearly variation is conceptualised to encompass the concepts of survival (due to all causes aside from hunting), reproduction, environmental variability, and demographic stochasticity; irresolvable variation, but can be low or high. Replicate variability simulates parameter uncertainty; resolvable through improved knowledge, and can be low or high. Lack of variation in *K* assumes that the fundamental carrying capacity of the system remains the same throughout the time period assessed.

We note that the use of a standard model framework with logistic growth, applied across a fast – slow gradient of species is a simplification, as density dependent (and depensatory) effects are likely to correlate with species position along this gradient (Stevens, Bence, Porter, & Parent, 2017; Williams, 2013). A potential modification of this to better capture differences in the fast – slow species gradient might be to use a generalised theta-logistic model, with a theta < 1 for ‘fast’ *r*-selected species, and a theta > 1 for ‘slow’ *K*-selected species. However while this is conceptually practical, such parameters are challenging to estimate in application, and there is likely to be evolutionary interactions on theta with experienced environmental variability (Williams, 2013). Furthermore, Sæther et al. (1996) show that environmental stochasticity can have a larger effect on optimal harvesting strategy than the form of density-dependence. Further caveats include that we do not explicitly consider Allee effects at low population sizes (Lacy & Pollak, J.P., 2020; Anderson et al. 2017), nor the full range of possible population dynamics (Saunders, Cuthbert, & Zipkin, 2018; Stevens et al., 2017; Williams, 2013). Different assumptions on the relationship between population growth rates and environmental variability are certainly possible (Colchero et al., 2019) and may induce feedbacks at a system level (Vilar & Rubi, 2018). We chose to use a simple logistic model also because of our focus on developing basic heuristics and cross-species comparisons: it provides comparability over our range of hypothetical species contexts using common, reasonable model assumptions.

This model assumes unstructured population dynamics with no spatial dynamics. As such, it ignores the impacts of age, sex, connectivity, and spatial structure in harvest systems (Colchero et al., 2019; Miller et al., 2019; Milner, Nilsen, & Andreassen, 2007; Youngflesh and Lynch 2017). Susceptibility of different age classes to environmental variability can have significant feedbacks on population growth rates, likely to be particularly important in species where juvenile conditions correlate with adult fertility (such as the ungulates we model here) (Colchero et al 2019). Sex biases in harvest often, but not always, increase the negative impacts of hunting on a population (Milner, Nilsen, & Andreassen, 2007). We also assume that populations are closed, which can accentuate population declines, and are thus a more precautionary approach to employ (Miller, Furness, Trinder, & Matthiopoulos, 2019), at least from the perspective of population persistence.

The **monitoring component** is simulated by a single variation factor (*m_i,t_*) acting on *N_i,t_*, to give an estimate of the population size ($\hat{N_{i,t}}$), to be used as the basis for management decisions.

(5) $\hat{N_{i,t}}=N_{i,t} (1+m_{i,t})$

where:

(6) $m_{i,t} \approx N(m_{i}^{m}, m_{i}^{sd})$

(7) $m_{i}^{m} \approx N(0, m^{sdm})$

(8) $m_{i}^{sd} \approx N(m^{msd}, m^{sdsd})$

We assume that$m^{sdm},m^{msd}, m^{sdsd}$ are constant for each species context and variation scenario, and ultimately monitoring variation has no systematic bias overall. Monitoring variation is conceptualised to encompass all the processes of sampling and observation, monitoring data analyses, and belief formation. Variation across years simulates inaccuracy or imprecision in monitoring; potentially resolvable with improved effort or monitoring technique, and can be low or high. Variation of mean bias over replications simulates parameter uncertainty regarding bias in monitoring; resolvable with improved knowledge regarding the monitoring methodology, and can be low or high. In reality, monitoring effectiveness is likely to vary with respect to the population size: with larger populations, monitoring is likely to miss or double count more individuals, and counts potentially rounded. However, we do not consider that monitoring small populations might result in larger proportional errors.

The **management decisions component** is partitioned into two parts. First, a harvest strategy is applied, converting $\hat{N_{i,t}}$into an initial quota, *Q_i,t_,* given a set of quota parameters (constants, *C1*, thresholds *T1,T2,* and proportions, *P1, P2*).

(9) $Q_{i,t}= \left\{ \begin{aligned} \\ C_{1}, \hat{N_{i,t}}\leq T_{1} \\ \hat{N_{i,t}}(P_{1}+\left( P_{2}- P_{1} \right)\left( \frac{\hat{N_{i,t}}-T_{2}}{T_{2}- T_{1}} \right), T_{1}\leq\hat{N_{i,t}}< T_{2} \\ P_{2}, T_{2}\leq\hat{N_{i,t}} \end{aligned} \right.$

Using this definition, we construct harvest strategies defined for constant, proportional, threshold proportional, with harvest proportions either stable or increasing with population size (see parameter sets in Table S1.2). We assume that the harvest strategies and the associated parameters remain consistent through the timeframe. This equation simulates evidence-based scientific recommendations of quota size (and is therefore not rounded to an integer at this stage).

*Q_i,t_* is then subject to random variation ($q_{i,t}$) to simulate the political interventions that often enter the quota setting process, to give a modified quota $Q_{i,t}^{'}$.

(10) $Q_{i,t}^{'}=Q_{i,t} (1+q_{i,t})$ (rounded to nearest non-negative integer)

(11) $q_{i,t} \approx N(0, q_{i}^{sd})$

Variability in the quota is designed to simulate the impacts of political processes on quota development, and can either not exist (management exactly follows scientific evidence) or can introduce a ‘high’ level of variability. We assume there is no parameter uncertainty in this case, and only allow variation over years (not replicates), and we assume no overall systematic bias in quota variation.

The **harvest implementation component** simulates imperfect harvest implementation, effected as a proportional variation (*h_i,t_*) around $Q_{i,t}^{'}$ to give the harvest (*H_i,t_*). This amount is then removed from *N_i,t_*.:

(12) $H_{i,t}=Q_{i,t}^{'} (1+h_{i,t})$ (rounded to nearest non-negative integer)

(13) $h_{i,t} \approx N(h_{i}^{m}, h_{i}^{sd})$

(14) $h_{i}^{m} \approx N(0, h^{sdm})$

(15) $h_{i}^{sd} \approx N(h^{msd}, h^{sdsd})$

(16) $N_{i,t+1}=N_{i,t}-H_{i,t}$

Variation across years simulates stochasticity in the harvest, and can be low or high. This variation can be conceptualised as both environmental stochasticity (irreducible) and user-driven imperfections (reducible, for example through increased enforcement or other incentive to achieve the quota), and therefore partly reducible overall. Variation across replications simulates parameter uncertainty in regards to the bias in harvest relative to the quota; resolvable through increased knowledge of the harvesters, and trust of the harvesters in the quota, and can be low or high. We simplify this by assuming that there is an unbiased estimate of how much will be harvested given a quota. In reality, hunting efficiency may vary with respect to the quota. For example, in both moose (Hunt, 2013) and ptarmigan (Eriksen, Moa, & Nilsen, 2018) hunting effectiveness increases at low tag numbers. Our formulation of the harvest imperfection as a coefficient of variance factor means that the variance will be smaller at smaller quota sizes.

A common conceptualisation of the functionality of a quota is to limit potential ‘tragedy of the commons’ by enforcing a limit on harvest, and this might be expected to produce a bias on harvest implementation such that it is more common for harvests to be below the quota than above. However, we note that in reality, quotas are often set in systems where the ‘total allowable catch’ or the maximum possible harvest legally possible under the set quota is much higher than the intended harvest (Bischof et al 2012). This is particularly common, for example, when quotas are specified with spatial or temporal specifications, or in terms of amounts per person. This means that decision makers need to estimate the relationship between the quota and the levels of harvest they intend to be taken (Moa, Eriksen, & Nilsen, 2017). Despite these critical assumptions, there are relatively few studies on imperfect harvest implementation in terrestrial wildlife systems (Bischof et al., 2012; Eriksen et al., 2018). In the current study, the quota and harvest components together define a system in which the quota is implemented with no systematic harvest bias. We conceptualize this quota as the ‘intended harvest’, or the amount expected to be harvested. We suggest that therefore the assumption that the simulated harvest may be normally distributed around the ‘intended harvest’ is reasonable, with the caveat it is unlikely to hold in all contexts.

We do not account for feedbacks and directional bias likely in harvest implementation (Eriksen et al., 2018; Hunt, 2013), and more generally through the harvest system (Bieg, McCann, & Fryxell, 2017; Fryxell, Packer, McCann, Solberg, & Sæther, 2010).

The **evaluation component** occurs after each simulation is complete, calculating the performance of each of the 1000 replicates over the entire timeframe, before summarising over the scenario run. Evaluation metrics are designed to reflect different potential objectives and stakeholder concerns, and cover a number of socio-ecological (i.e. population-based) and harvest-based sustainability objectives (Table 1). These were gathered from both theoretical literature and empirical collations of harvest objectives stated in wildlife harvest policy documents (e.g. Artelle et al. 2018), and reduced to those feasible given the data generated by the model, without the need for additional subjective valuation metrics.

As multiple sustainability metrics may be relevant to a context, we develop a number of composite metrics illustrating potentially common sets of metrics simulating different stakeholder perspectives or sets of stakeholder values needing to be considered by a decision-maker in a particular context (Table 2). To make scores comparable across harvest systems for each environmental context, we scale individual metrics so that all range from 0 to 100, with more desirable outcomes gaining the highest scores. Conversions to effect this are shown in Table S1.1, with original metric limits in Table S1.2. Scaling is applied to all replicates, prior to summarising expected values (risk neutral, as a mean score) for individual metrics per harvest decision run (i.e. set of harvest system and harvest parameters). Due to co-dependency between individual metrics, composite metric scores are calculated per replicate, before also being summarised to the expected value over each run. This scaling and aggregating protocol makes several assumptions regarding the equivalencies of value within and among individual metrics. Specifically, using the scaled scores for the individual metrics assumes these are directly related to utility, and imposes a linear preference assumption, which treats for example, gains from 0-10% as equivalent to gains from 90-100%. Aggregation of the different individual metrics by averaging into composite metrics assumes these benefits are equivalent and substitutable, and further, that Utilitarian “aggregate good” ethics are appropriate should different benefits accrue to different stakeholders.

In summary, we assume that there can be yearly variation in *r, m, q,* and *h,* and variation over replications for *r, m,* and *h.* We assume variability in *r, m,* and *h,* can be low or high, simulating partial resolvability of these phenomena. We assume variability in *q* can be zero or high. In all the variable parameters, we assume normal distributions (as specified in the above equations, using the species specific parameters given in section S1.2), with no correlation of error. We select normal distributions as we assume the variability is due to a number of different sources, and the central limit theorem would suggest that these might coalesce to a normal distribution. In the monitoring, quota, and harvest variation, for simplicity we assume a coefficient of variation function proportional to the population or quota. We highlight that each component of the model remains highly simplified in this analysis, and intend this to provide a simplified baseline study, onto which further developments can be built.

We made no attempt to value the monetary aspects of harvest systems (Gren et al., 2018), nor implementation costs (Kritzer et al., 2019). We note that in our analysis, by limiting the time series to 20 years but otherwise not adjusting for time discounting, we effectively default to a zero discount until year 20, and full discount thereafter. The consequences of this are that maximizing harvest objectives can drive populations to undesirably low levels when not checked by other objectives or inherent risk of variability in the species context. This results in some scenarios – particularly noticeable in the constant harvest strategy for the ‘slow’ species – resulting in the objective of maximizing harvest (without any other constraints) causing a draw down on the population to the point at which harvests are limited by the population size (often extinction). While this is not an acceptable scenario in any definition of ‘sustainability’ we use it as a cautionary note as to what focus on certain metrics and ignorance of others may cause. Applying time discounting across the time series is likely to further increase this (undesirable) effect, even with infinite time horizons (Lande et al., 1994), as they would place more value on larger harvests in the earlier points in the time frame, and discount smaller harvests caused by population decline in later years. There is no universally applicable method for defining appropriate discount rates for non-monetary values (Botzen & van den Bergh, 2014), but here we note that despite the absence of a time discounting procedure, the limited time frame of assessment effects this phenomenon in this case.

As we are not searching for equilibria, we do not apply a ‘burn-in’ time period, but rather start the population from the initial population given by the species parameters. We also do not consider time lags in management decisions, which can be common particularly in low-knowledge scenarios (Manning, Stevens, & Williams, 2019).

We also assume no other temporal feedbacks aside from those effected by density dependence and application of the harvest strategy to generate the initial quota. However, these may be common features of management systems, for example populations may be more prone to environmental variability under high population densities, and harvesters and managers may react systematically to different population densities and quotas (Bieg, McCann, & Fryxell, 2017; Fryxell, Packer, McCann, Solberg, & Sæther, 2010).

### Figure S1.1: Overall MSE model framework


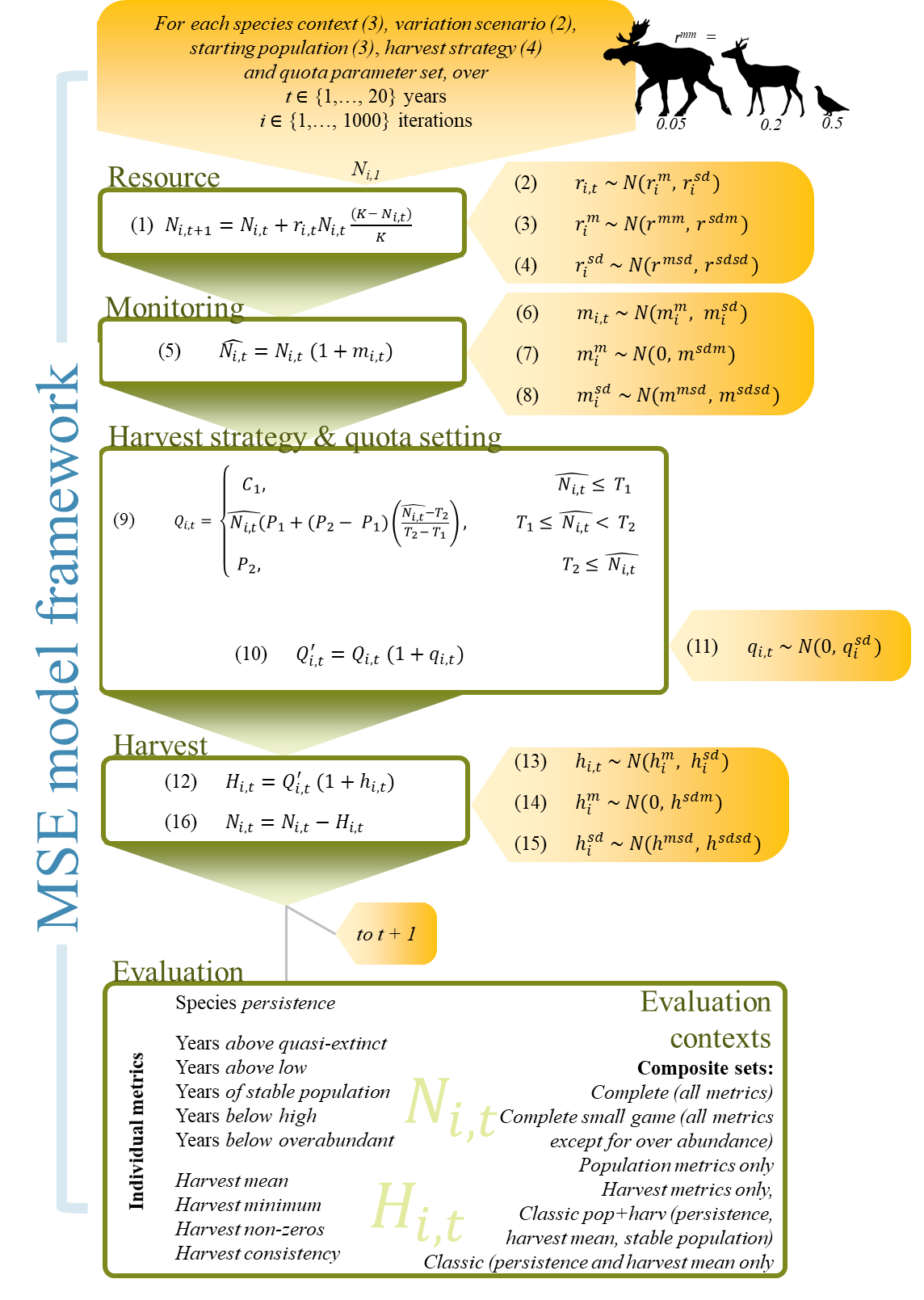


### Table S1.1: Evaluation framework: scaling of individual metrics.

Original individual metrics as calculated during the simulation have either defined or undefined theoretical limits. *max** indicates an undefined limit, and the limit is taken as the maximum observed value within each environmental context (i.e. species, variability, and starting population scenario). Transformation and scaling converted these original metrics onto a scale of 0:100, where 0 represents no achievement of that score (for defined endpoints) or the worst case observed (for undefined limits, e.g. in Harvest variability) and 100 represents the highest possible score or best case achieved.

| **Original metric** | **Theoretical limit** | | **Description of limit** | **Transformation rule**  Start → end | **Transformed metric** |
| --- | --- | --- | --- | --- | --- |
| Population persistence | min | 0 | no extinction within timeframe | Negate + scale  0 → 100  1 → 0 | Population persistence |
|  | max | 1 | extinction within timeframe |  |  |
| Quasi-extinction | min | 0 | all years satisfactory | Negate + scale  0 → 100  20 → 0 | Above quasi-extinction |
|  | max | 20 | all years not satisfactory |  |  |
| Low population | min | 0 | all years satisfactory | Negate + scale  0 → 100  20 → 0 | Above low |
|  | max | 20 | all years not satisfactory |  |  |
| Stable population | min | 0 | no years satisfactory | Scale  0 → 0  20 → 100 | Stable population |
|  | max | 20 | all years satisfactory |  |  |
| High population | min | 0 | all years satisfactory | Negate + scale  0 → 100  20 → 0 | Below high |
|  | max | 20 | all years not satisfactory |  |  |
| Overabundance | min | 0 | all years satisfactory | Negate + scale  0 → 100  20 → 0 | Below overabundant |
|  | max | 20 | all years not satisfactory |  |  |
| Harvest mean | min | 0 | mean harvest of zero | Scale  0 → 0  *max* → 100 | Harvest mean |
|  | max | *max** | maximum mean harvest observed |  |  |
| Harvest min | min | 0 | minimum harvest of zero | Scale  0 → 0  *max* → 100 | Harvest min |
|  | max | *max** | largest minimum harvest observed |  |  |
| Harvest zeros | min | 0 | No years with zero harvest | Negate + scale  0 → 100  20 → 0 | Harvest zeros |
|  | max | 20 | All years with zero harvest |  |  |
| Harvest variability | min | 0 | No harvest variability | Negate + scale  0 → 100  *max* → 0 | Harvest variability |
|  | max | *max** | High harvest variability |  |  |

## Table S1.2 Limits of original metrics

Limits of original individual metrics as calculated during the simulation for each environmental context.

| **Original metric** | **Theoretical limit** | | **Outcome** | **Moose** | | | | | | **RoeDeer** | | | | | | | | **Ptarmigan** | | | | | |
| --- | --- | --- | --- | --- | --- | --- | --- | --- | --- | --- | --- | --- | --- | --- | --- | --- | --- | --- | --- | --- | --- | --- | --- |
|  |  |  |  | **SID1** | **SID2** | **SID3** | **SID4** | **SID5** | **SID6** | | **SID1** | **SID2** | **SID3** | **SID4** | **SID5** | **SID6** | **SID1** | | **SID2** | **SID3** | **SID4** | **SID5** | **SID6** |
| Population persistence | min | 0 | Best | 0 | 0 | 0 | 0 | 0 | 0 | | 0 | 0 | 0 | 0 | 0 | 0 | 0 | | 0 | 0 | 0 | 0 | 0 |
|  | max | 1 | Worst | 1 | 1 | 1 | 1 | 1 | 1 | | 1 | 1 | 1 | 1 | 1 | 1 | 1 | | 1 | 1 | 1 | 1 | 1 |
| Quasi-extinction | min | 0 | Best | 0 | 0 | 0 | 0 | 0 | 0 | | 0 | 0 | 0 | 0 | 0 | 0 | 0 | | 0 | 0 | 0 | 0 | 0 |
|  | max | 20 | Worst | 17 | 18 | 19 | 19 | 16 | 18 | | 16 | 19 | 19 | 19 | 14 | 19 | 18 | | 19 | 19 | 19 | 17 | 19 |
| Low population | min | 0 | Best | 0 | 0 | 20 | 15 | 0 | 0 | | 0 | 0 | 9 | 8 | 0 | 0 | 0 | | 0 | 1 | 1 | 0 | 0 |
|  | max | 20 | Worst | 19 | 19 | 20 | 20 | 18 | 18 | | 19 | 19 | 20 | 20 | 17 | 19 | 19 | | 19 | 20 | 20 | 18 | 19 |
| Stable population | min | 0 | Worst | 1 | 1 | 0 | 0 | 0 | 0 | | 1 | 1 | 0 | 0 | 0 | 0 | 1 | | 1 | 0 | 0 | 0 | 0 |
|  | max | 20 | Best | 20 | 20 | 0 | 5 | 19 | 19 | | 20 | 20 | 11 | 12 | 19 | 19 | 20 | | 20 | 19 | 19 | 19 | 19 |
| High population | min | 0 | Best | 0 | 0 | 0 | 0 | 1 | 1 | | 0 | 0 | 0 | 0 | 1 | 1 | 0 | | 0 | 0 | 0 | 1 | 1 |
|  | max | 20 | Worst | 13 | 15 | 0 | 0 | 20 | 20 | | 18 | 18 | 6 | 9 | 20 | 20 | 19 | | 19 | 17 | 18 | 20 | 20 |
| Over-abundance | min | 0 | Best | 0 | 0 | 0 | 0 | 0 | 0 | | 0 | 0 | 0 | 0 | 0 | 0 | 0 | | 0 | 0 | 0 | 0 | 0 |
|  | max | 20 | Worst | 6 | 11 | 0 | 0 | 19 | 19 | | 15 | 16 | 3 | 7 | 19 | 19 | 18 | | 18 | 15 | 17 | 19 | 19 |
| Harvest mean | min | 0 | Worst | 0 | 0 | 0 | 0 | 0 | 0 | | 0 | 0 | 0 | 0 | 0 | 0 | 0 | | 0 | 0 | 0 | 0 | 0 |
|  | max | *n* | Best | 52 | 64 | 8 | 13 | 94 | 121 | | 1894 | 2998 | 803 | 1304 | 3121 | 4349 | 12328 | | 14786 | 9706 | 16027 | 13184 | 20063 |
| Harvest min | min | 0 | Worst | 0 | 0 | 0 | 0 | 0 | 0 | | 0 | 0 | 0 | 0 | 0 | 0 | 0 | | 0 | 0 | 0 | 0 | 0 |
|  | max | *n* | Best | 49 | 49 | 4 | 5 | 93 | 95 | | 1581 | 1669 | 44 | 47 | 2969 | 3244 | 7641 | | 7366 | 2331 | 2490 | 12746 | 16004 |
| Harvest zeros | min | 0 | Best | 0 | 0 | 0 | 0 | 0 | 0 | | 0 | 0 | 0 | 0 | 0 | 0 | 0 | | 0 | 0 | 0 | 0 | 0 |
|  | max | 20 | Worst | 20 | 20 | 20 | 20 | 20 | 20 | | 20 | 20 | 20 | 20 | 20 | 20 | 20 | | 20 | 20 | 20 | 20 | 20 |
| Harvest variability | min | 0 | Best | 0 | 0 | 0 | 0 | 0 | 0 | | 0 | 0 | 0 | 0 | 0 | 0 | 0 | | 0 | 0 | 0 | 0 | 0 |
|  | max | *s* | Worst | 111 | 152 | 32 | 58 | 188 | 212 | | 2082 | 3017 | 1632 | 2056 | 3122 | 4656 | 6934 | | 12132 | 6657 | 10824 | 8008 | 13791 |

## S1.2 Hypothetical species and parameters

We develop cases based on three hypothetical species spanning a range of common game species: the moose (*Alces alces*), roe deer (*Capreolus capreolus*), and willow ptarmigan (*Lagopus lagopus*)(Table S1.1). We loosely base these hypothetical species on wildlife species harvested in a Norwegian context. To provide consistency between species, overall variation in the growth rate is specified to be equal to the species growth rate in the high variability scenario, and half of the species growth rate in the low variation scenario. For each variable parameter (here generalised to *x*), total variation (*x^TSD^*) is split between replications and years, by partitioning the overall standard deviation equally into the replication level standard deviation (that determines the vector of the parameter over the years, *x^sdm^*), and the scenario level mean standard deviation (that determines the parameter mean value over the replications, *x^msd^*).

(17) $x^{msd}=x^{sdm}=\frac{x^{TSD}}{2}$

The standard deviation of the replication level standard deviation (*x^sdsd^*)was defined at 1/3 of the standard deviation of the mean standard deviation. Simulations of $x_{i}^{sd}$were truncated to remain positive, at a minimum of 0.0001.

(18) $x^{sdsd}=\frac{x^{sdm}}{3}$

The moose (*Alces alces*) is a large ungulate assumed to have a relatively low growth rate, carrying capacity, monitoring variation, and critical thresholds (Table S1.1). A description of moose population and harvest dynamics in a Scandinavian context is available in Sæther et al. (2001).

The roe deer (*Capreolus capreolus*) is a small ungulate with a moderate growth rate, carrying capacity, monitoring variation, and critical thresholds. A description of roe deer population and harvest dynamics is available in Andersen et al. (1998).

The willow ptarmigan (*Lagopus lagopus*) is a game bird with a relatively large potential growth rate, carrying capacity, monitoring variation, and critical thresholds. A description of willow ptarmigan population and harvest dynamics in a Scandinavian context is available in Eriksen et al. (Eriksen et al., 2018).

From these parameters, we can calculate the standard maximum sustainable yield (*MSY*) conditions given no stochasticity, occurring at *K*/2, and with an annual harvest of *rK*/4 under the logistic growth assumption (with values rounded to the nearest integer). For the moose, MSY is expected at a population of 1112 (notably larger than the moderate starting population, the high critical threshold, and close to the overabundant critical threshold), allowing an annual harvest of 28 individuals. For the roe deer, MSY is expected at a population size of 13900 (also larger than the moderate starting population, and the high critical threshold), allowing a harvest of 1390 individuals. For the ptarmigan, MSY is expected at 30000 (also larger than the moderate starting population, and the high critical threshold) with a harvest of 7500. We provide these calculations for comparison only: MSY using these calculations is a theoretical construct under strict assumptions and will often overestimate the true maximum sustainable yield (Quinn & Collie, 2005). We also note that, given the parameters used, the MSY population level is often higher than desirable for other stakeholder concerns, particularly in the ungulate systems.

### Table S1.1: Species parameters and variable parameter assumptions

We defined three species contexts, which specified the value of fixed constants for mean *r, K,* critical thresholds, and starting populations, and the level of variations deemed low and high for *r* and *m.* While these are loosely based on real species, the values are specified to facilitate scenario comparisons. We also provide here parameters used for quota and harvest variability, assumed to be equal across the species gradient. Variable parameters are given as the overall mean (*x^mm^*) and overall standard deviation (*x^TSD^*) for low and high variation scenarios; a description of how these are partitioned into yearly and replication level distribution parameters is provided in section S1.2.

| **Component** | **Parameter – variation scenario** | | **Moose**  (*Alces alces*) | | **Roe deer**  (*Capreolus capreolus*) | | **Ptarmigan**  (willow ptarmigan, *Lagopus lagopus*) | |
| --- | --- | --- | --- | --- | --- | --- | --- | --- |
|  |  |  | ***Mean***  ***x^mm^*** | ***SD***  ***x^TSD^*** | ***Mean***  ***x^mm^*** | ***SD***  ***x^TSD^*** | ***Mean***  ***x^mm^*** | ***SD***  ***x^TSD^*** |
| Resource | *r* | low  high | 0.05 | 0.025  0.05 | 0.2 | 0.1  0.2 | 0.5 | 0.25  0.5 |
|  | *K* | | 2225 | | 27800 | | 60000 | |
| Monitoring | *m* | low  high | 0 | 0.05  0.15 | 0 | 0.1  0.3 | 0 | 0.15  0.45 |
| Quota | *q* | none  high | 0 | 0  0.1 | 0 | 0  0.1 | 0 | 0  0.1 |
| Harvest | *h* | low  high | 0 | 0.05  0.25 | 0 | 0.05  0.25 | 0 | 0.05  0.25 |
| Evaluation critical thresholds | *Extinction*  *Quasi-extinction*  *Low*  *High*  *Overabundant* | | 1  60  300  900  1200 | | 1  167  2780  11120  19460 | | 1  5000  10000  25000  40000 | |
| Starting populations (respective to critical thresholds) | *Moderate start*  *Quasi-extinct start*  *Overabundant start* | | 600  60  1200 | | 6950  167  19460 | | 17500  5000  40000 | |

## S1.3 Harvest strategies, quota parameters and optimization

Harvest strategies analysed include ‘*constant*’ (a set number of individuals harvested yearly), ‘*proportional*’ (a set proportion of the population harvested yearly), ‘*threshold proportional’* (a set proportion taken yearly, provided the population is above a certain threshold), and ‘*no harvest*’. These harvest strategies are defined by the quota parameters that define constants, thresholds, and proportions (Table S1.2).

Harvest strategies (also known as harvest control rules) can be either strictly followed to develop quotas, or form the principles behind quota setting (Kvamsdal et al., 2016). Here we assume the former (through eq. 9) although allow some flexibility for adjustment (through eq. 10). These harvest strategies are variably termed in the literature. Some examples:

- *Constant*: fixed-quota, constant catch (Deroba & Bence, 2008).
- *Proportional*: constant mortality rate, constant-*F*, this is one of the most commonly used rules in fisheries, often suggested to be optimal with perfect information (Deroba & Bence, 2008).
- *Threshold*-*proportional*: proportional threshold, developed specifically for stochastic & uncertain contexts (Engen, Lande, & Sæther, 1997); also called ‘threshold’ by some fisheries sources (Deroba & Bence, 2008). Note in this case, we apply the proportion with respect to the whole population, if above a threshold (c.f. applying it to the proportion of the population above the threshold).

Other rules not examined here include other variations on threshold-based rules, including constant escapement (100% take above a threshold), increasing rates above or decreasing rates below a threshold, conditional constant catch (constant amount, unless removing that amount would exceed some predetermined maximum mortality rate) with variations on this including no take below the threshold, proportional take below the threshold. The intentions for the various rules including those not utilised here are summarised in (Deroba & Bence, 2008). Of note, the harvest strategies we analyse here are focused on the population dynamics within a system, as we do not consider the relative monetary costs and benefits of harvesting. Further harvest strategies including the monetary economics of harvesting are possible (Kvamsdal et al., 2016).

Harvest rules implemented for small game birds (grouse species) in Europe and North America are reviewed in (Moa et al., 2017). They note that proportional and threshold-proportional principles are common, however in practice bag sizes are often relatively more limited at large population sizes, against recommendations (Moa et al., 2017).

Each harvest strategy can be utilised with different harvest parameters, and it is this combination (of harvest strategy and harvest parameters) that forms the main ‘decision variables’ in the MSE model. To sample possible harvest parameter options, we employed either a stopping rule or a grid search method, incrementally varying the parameters across the option space (Table S1.2). This search method does not cover the entire option space defined, but represents a pragmatic approach towards illustrating trade-offs across the parameter space, and optimization in relevant parameter space given the volume of parameter options available, and given the likelihood of multiple optima. While this might result in fine details of the comparisons being inaccurate, we expect the main conclusions to hold, as we saw no severe gaps in the trends across the parameter space (see Main text and Supporting Information S2).

### Table S1.2: Harvest strategies, quota parameters, and heuristics for searching the option space.

Initial harvest quotas are developed such that (as defined in eq. 9) the constant, *C1*, applies from a population of 0 until the threshold *T1*. The proportion *P1* then applies, linearly transitioning to *P2* at the threshold *T2*. After this threshold, the proportion continues at *P2*. With this same set of equations, we can define the *constant, proportional,* and *threshold- proportional* harvest strategies (and allow for development to threshold-increasing-proportional systems). We simulate over a range (option space) of harvest parameters for each harvest strategy. No harvest was implemented simply as zero harvest.

| **Harvest strategy** | **Harvest parameters (option space searched)** | | | | |
| --- | --- | --- | --- | --- | --- |
|  | **C1** | **P1** | **P2** | **T1** | **T2** |
| *Constant* | 0 : stop; Increase in increments of 1% of moderate staring population; Stop if probability of non-extinction = 0 | 0 | 0 | Inf | Inf |
| *Proportional* | 0 | 0.01 : 0.50; Increase in increments of 0.01 | = P1 | 0 | Inf |
| *Threshold-proportional* | 0 | 0.01 : 0.50; Increase in increments of 0.01 | = P1 | From quasi-extinction critical threshold : moderate starting population size for each species; increase in increments of 1% of (moderate starting population – quasi-extinction) | Inf |

## S1.4 Scenarios and comparisons

To examine the performance of the harvest strategies, we first focused on comparing results for each species context and harvest strategy for scenarios where all variability in *r,* *m, q*, and *h* were either all low (*low variability*) or all high (*high variability*), and starting populations were at the midpoint of low and high critical thresholds (*moderate starting population*). This means that the magnitude of uncertainty was correlated between the components, however the pattern of uncertainty across years was random for all components. We then repeated the simulations with populations starting at quasi extinction (*low starting population*), and populations starting at overabundance (*high starting population*), to examine the robustness of the harvest strategies to extreme perturbations in population size and the recovery potential in such cases. Such simulations are also relevant for special management cases, for example harvest of an overabundant invasive species, or recovery of endangered species into harvestable populations. Simulations were run such that each harvest strategy and harvest parameter variation is run with exactly the same starting and variable conditions (*r,* *m, q*, *h*) under each respective scenario (species context, variability level, and starting population size) combination.

We tested both outcomes based on the ‘true’ simulated populations *N_i,t_*, as well as metrics based on the simulated monitoring data ($\hat{N_{i,t}}$), but as the latter were virtually identical to the former in this case (as might be expected with normal distributions on errors) we report only on *N_i,t_*.

In this analysis, we focus on the implications of alternative harvest strategies and sustainable metrics, and therefore only test the cases of ‘low’ and ‘high’ variability for each hypothetical species, and do not resolve here which sources of variability or uncertainty are most influential or valuable to address (Canessa et al., 2015; Davis, Chadès, Rhodes, & Bode, 2019).

Scenarios 3 and 4 simulate recovery of populations from quasi-extinction levels, at *low* and *high* *variability* scenarios respectively, via the use of a single harvest strategy set quota parameters across the entire time-frame. The *moose* was largely unable to reach a *stable population* level, even with zero harvest, for any variability scenario. This is not unexpected given the mean population growth rate specified for this species (r = 0.05) under the given time frame (20 years), and the critical thresholds specified (from a starting point of quasi-extinction = 60, population growth without harvest would be expected to increase the population to 159 (60 x (1 + 0.05)^20^ ), which remains below the low critical threshold = 300). There could be a higher level of recovery of *moose* above the *quasi-extinction* critical threshold for the more complex harvest strategies, and interestingly the high variation scenario performed considerably better than the low variation scenario in this regard, because more replicates received higher population growth rates, while thresholds minimized losses. The *roe deer* showed more recovery, holding *stable population* for around four years, across all harvest strategies and variation scenarios. The *stable population* outcomes were quite variable, however, and while the high variability scenario achieved all years at above the *quasi-extinction* critical threshold, the higher variability scenario achieved suboptimal scores with high levels of variability. The *ptarmigan*, with a much higher rate of population growth on average, would be expected to attain a better recovery, and had *stable population* scores only slightly lower than the baseline moderate start population scenario. The lower constant harvest rate needed to effect this increased the *above quasi-extinct* scores for this harvest strategy, but with a corresponding likely decline in *below high*.

Scenarios 5 and 6 simulate harvesting of populations starting at overabundant levels, at *low* and *high* *variability* scenarios respectively, via the use of a single harvest strategy set quota parameters across the entire time-frame. Results were similar to the baseline scenarios, albeit with higher *harvest mean* and lower *stable population* scores, particularly for the slower-larger species with the effect declining for *ptarmigan*. Typically differences manifested in compatibility sets being classified as high populations rather than stable populations, in both the *moose* and *roe deer*.

### Table S1.3: Scenarios, applied for each species and harvest procedure

| **Scenario description** | **Scenario ID (SID)** | **Starting population size (*N_0_*)** | ***Replicate level variability in mean:*** | | | ***Yearly variation in:*** | | | |
| --- | --- | --- | --- | --- | --- | --- | --- | --- | --- |
|  |  |  | **Reproductive rate (*r*)** | **Monitoring (*m*)** | **Harvest (*h*)** | **Reproductive rate (*r*)** | **Monitoring (*m*)** | **Quota (*q*)** | **Harvest (*h*)** |
| Low variation – moderate starting population | 1 | mid | low | low | low | low | low | none | low |
| High variation – moderate starting population | 2 | mid | high | high | high | high | high | high | high |
| Low variation – low starting population | 3 | low | low | low | low | low | low | none | low |
| High variation – low starting population | 4 | low | high | high | high | high | high | high | high |
| Low variation – high starting population | 5 | high | low | low | low | low | low | none | low |
| High variation – high starting population | 6 | high | high | high | high | high | high | high | high |

## References

Andersen, R., Duncan, P., & Linnell, J. D. C. (1998). *The European roe deer: the biology of success*. Scandinavian University Press.

Anderson, S. C., Branch, T. A., Cooper, A. B., & Dulvy, N. K. (2017) Black-swan events in animal populations. *Proceedings of the National Academy of Sciences, 114* (12) 3252-325. doi: 10.1073/pnas.1611525114

Bieg, C., McCann, K. S., & Fryxell, J. M. (2017). The dynamical implications of human behaviour on a social-ecological harvesting model. *Theoretical Ecology*, *10*(3), 341–354. doi: 10.1007/s12080-017-0334-3

Bischof, R., Nilsen, E. B., Brøseth, H., Männil, P., Ozoliņš, J., & Linnell, J. D. C. (2012). Implementation uncertainty when using recreational hunting to manage carnivores. *Journal of Applied Ecology*, *49*(4), 824–832. doi: 10.1111/j.1365-2664.2012.02167.x

Botzen, W. J. W., & van den Bergh, J. C. J. M. (2014). Specifications of Social Welfare in Economic Studies of Climate Policy: Overview of Criteria and Related Policy Insights. *Environmental and Resource Economics*, *58*(1), 1–33. doi: 10.1007/s10640-013-9738-8

Canessa, S., Guillera-Arroita, G., Lahoz-Monfort, J. J., Southwell, D. M., Armstrong, D. P., Chadès, I., … Converse, S. J. (2015). When do we need more data? A primer on calculating the value of information for applied ecologists. *Methods in Ecology and Evolution*, *6*(10), 1219–1228. doi: 10.1111/2041-210X.12423

Davis, K. J., Chadès, I., Rhodes, J. R., & Bode, M. (2019). General rules for environmental management to prioritise social ecological systems research based on a value of information approach. *Journal of Applied Ecology*, *56*(8), 2079–2090. doi: 10.1111/1365-2664.13425

Deroba, J. J., & Bence, J. R. (2008). A review of harvest policies: Understanding relative performance of control rules. *Fisheries Research*, *94*(3), 210–223. doi: 10.1016/j.fishres.2008.01.003

Engen, S., Lande, R., & Sæther, B.-E. (1997). Harvesting Strategies for Fluctuating Populations Based on Uncertain Population Estimates. *Journal of Theoretical Biology*, *186*(2), 201–212. doi: 10.1006/jtbi.1996.0356

Eriksen, L. F., Moa, P. F., & Nilsen, E. B. (2018). Quantifying risk of overharvest when implementation is uncertain. *Journal of Applied Ecology*, *55*(2), 482–493. doi: 10.1111/1365-2664.12992

Fryxell, J. M., Packer, C., McCann, K., Solberg, E. J., & Sæther, B.-E. (2010). Resource Management Cycles and the Sustainability of Harvested Wildlife Populations. *Science*, *328*(5980), 903–906. doi: 10.1126/science.1185802

Hunt, L. M. (2013). Using human-dimensions research to reduce implementation uncertainty for wildlife management: a case of moose (Alces alces) hunting in northern Ontario, Canada. *Wildlife Research*, *40*(1), 61–69. doi: 10.1071/WR12185

Kvamsdal, S. F., Eide, A., Ekerhovd, N.-A., Enberg, K., Gudmundsdottir, A., Hoel, A. H., … Vestergaard, N. (2016). Harvest control rules in modern fisheries management. *Elem Sci Anth*, *4*(0), 000114. doi: 10.12952/journal.elementa.000114

Manning, S. E., Stevens, B. S., & Williams, D. M. (2019). Simulated performance of multi-year harvest regulation cycles for wild turkeys. *The Journal of Wildlife Management*, *83*(5), 1032–1042. doi: 10.1002/jwmg.21678

Miller, J. A. O., Furness, R. W., Trinder, M., & Matthiopoulos, J. (2019). The sensitivity of seabird populations to density-dependence, environmental stochasticity and anthropogenic mortality. *Journal of Applied Ecology*, *56*(9), 2118–2130. doi: 10.1111/1365-2664.13448

Milner, J. M., Nilsen, E. B., & Andreassen, H. P. (2007). Demographic side effects of selective hunting in ungulates and carnivores. *Conservation Biology: The Journal of the Society for Conservation Biology*, *21*(1), 36–47. doi: 10.1111/j.1523-1739.2006.00591.x

Moa, P. F., Eriksen, L. F., & Nilsen, E. B. (2017). Harvest Regulations and Implementation Uncertainty in Small Game Harvest Management. *Frontiers in Ecology and Evolution*, *5*. doi: 10.3389/fevo.2017.00118

Quinn, T. J., & Collie, J. S. (2005). Sustainability in single-species population models. *Philosophical Transactions of the Royal Society of London. Series B, Biological Sciences*, *360*(1453), 147–162. doi: 10.1098/rstb.2004.1577

Sæther, B.-E., Engen, S., & Lande, R. (1996). Density-Dependence and Optimal Harvesting of Fluctuating Populations. *Oikos*, *76*(1), 40–46. JSTOR. doi: 10.2307/3545746

Sæther, B.-E., Engen, S., & Solberg, E. J. (2001). Optimal harvest of age-structured populations of moose Alces alces in a fluctuating environment. *Wildlife Biology*, *7*(3), 171–179. doi: 10.2981/wlb.2001.021

Stevens, B. S., Bence, J. R., Porter, W. F., & Parent, C. J. (2017). Structural uncertainty limits generality of fall harvest strategies for wild turkeys. *The Journal of Wildlife Management*, *81*(4), 617–628. doi: 10.1002/jwmg.21228

Williams, C. K. (2013). Accounting for wildlife life-history strategies when modeling stochastic density-dependent populations: A review. *The Journal of Wildlife Management*, *77*(1), 4–11. doi: 10.1002/jwmg.429

Youngflesh, C. & Lynch, H. J. (2017) Population persistence and black-swan events. *Proceedings of the National Academy of Sciences*, *114* (43) E8953-E8954. doi: 10.1073/pnas.1713621114
