# Supplementary material for: Heuristics for the sustainable harvest of wildlife in stochastic social-ecological systems
Source: PLoS One. 2021 Nov 19;16(11):e0260159. doi: 10.1371/journal.pone.0260159 (PMC8604319; doi:10.1371/journal.pone.0260159)

## Supporting information S2: Additional results

Accompanying manuscript: Heuristics for the sustainable harvest of wildlife in stochastic social-ecological systems.

Authors: Elizabeth Law, John D. C. Linnell, Bram van Moorter, Erlend B. Nilsen.

Six environmental scenarios (i.e. combination of starting population size and level of uncertainty/variability) were defined for each of the three species. Scenario ID (SID) codes can be found in Figure 1, and fully described in Table S1.3. Individual metrics are defined in Table 1, and composite metrics in Table 2. As the constant harvest levels differ due to population sizes of the various species, we have scaled these with respect to their respective ‘moderate’ population size. Simulations for constant harvests iterated over increasing constant harvest parameters until all the replicate iterations resulted in population sizes of zero at the end of the time period (i.e. probability of persistence equal to zero; see methods detail). As such, their values do not necessarily span the whole width of the facet window. This is particularly evident under small starting population sizes (SID 3-4) where the small population size effectively limits the possibility of harvesting without driving the population to extinction.

## S2.1 Individual and composite metric scores across decision variables, with variability

### Figure S2.1.1 Individual metrics, with variability

Expected (mean) scores, median scores, and the 95% Credible Interval for individual metrics for each species (colour), across the simulated harvest parameters (x-axis) for each harvest strategy (panel columns), and each individual metric (panel rows). The threshold-proportional strategy is represented by 5 threshold values evenly sampling across the range of thresholds tested. Separate sets are given for each environmental context scenario (SID), as indicated by the set titles.


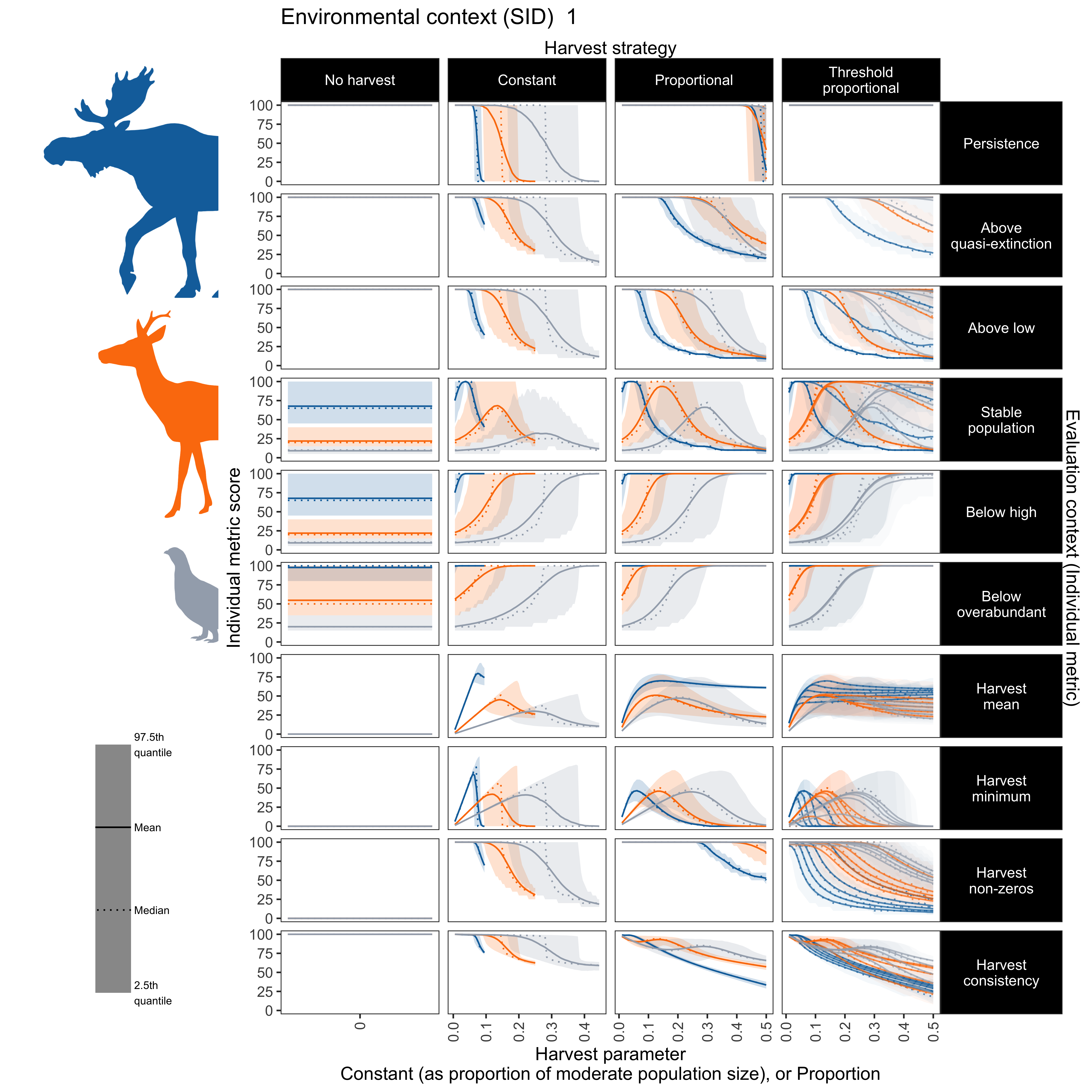


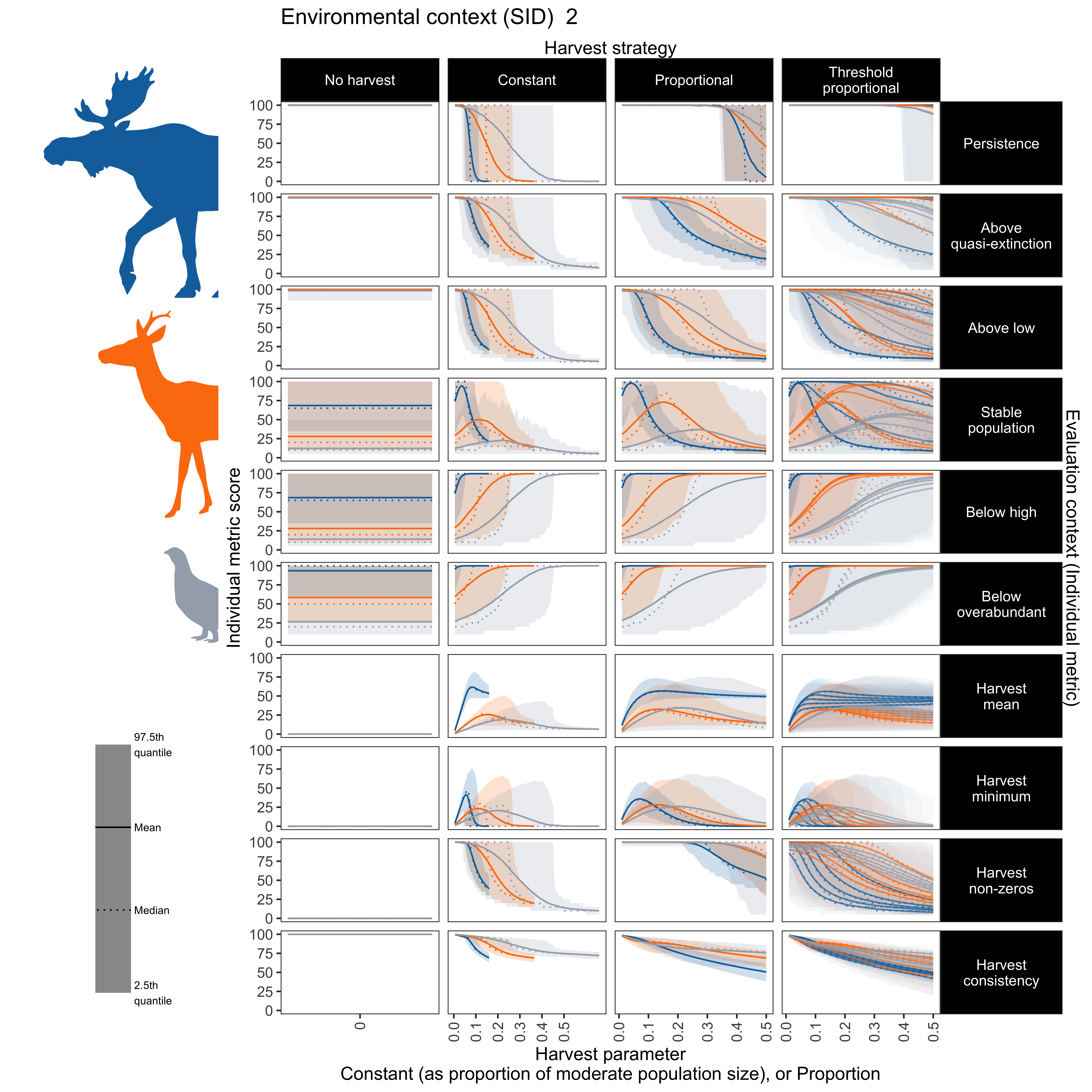


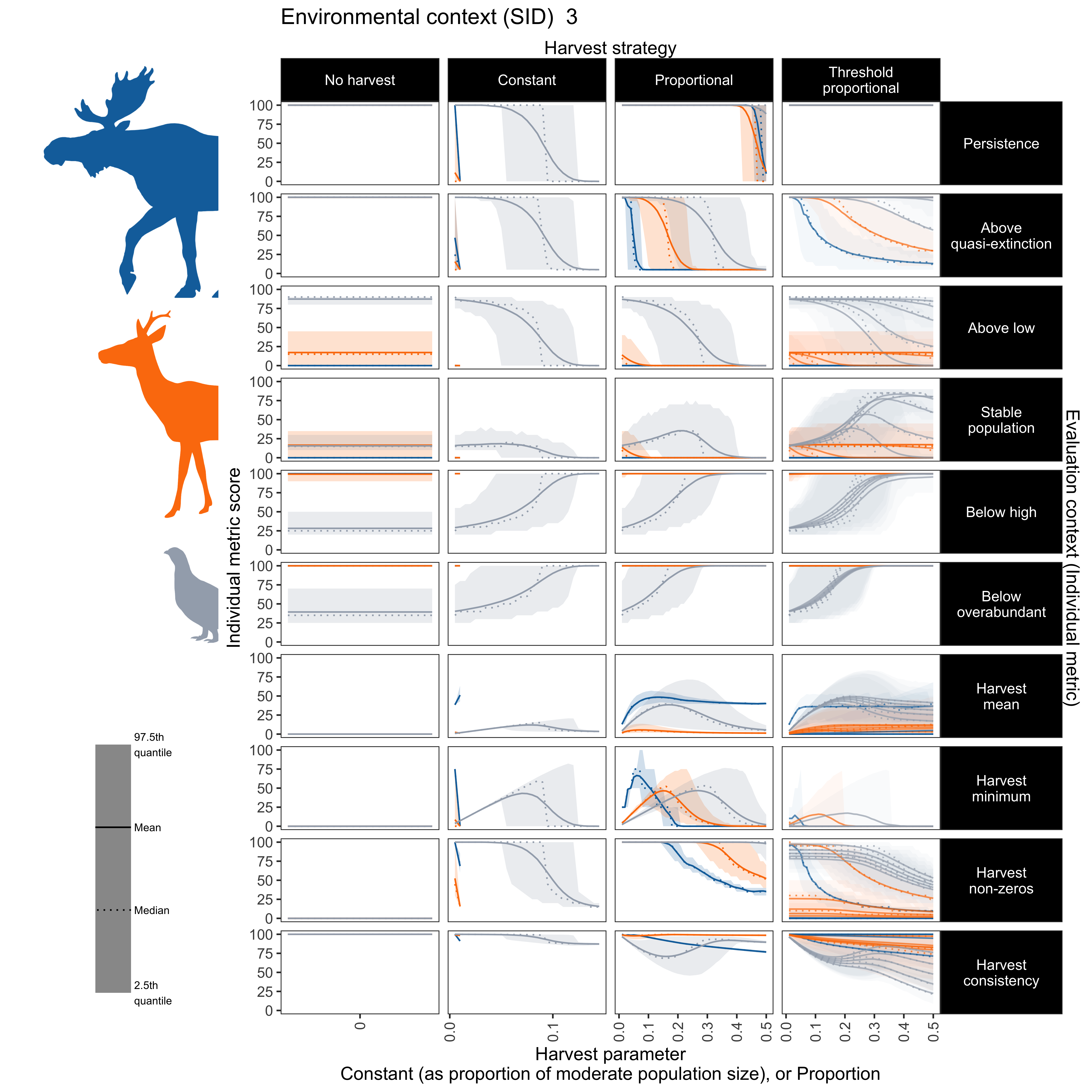


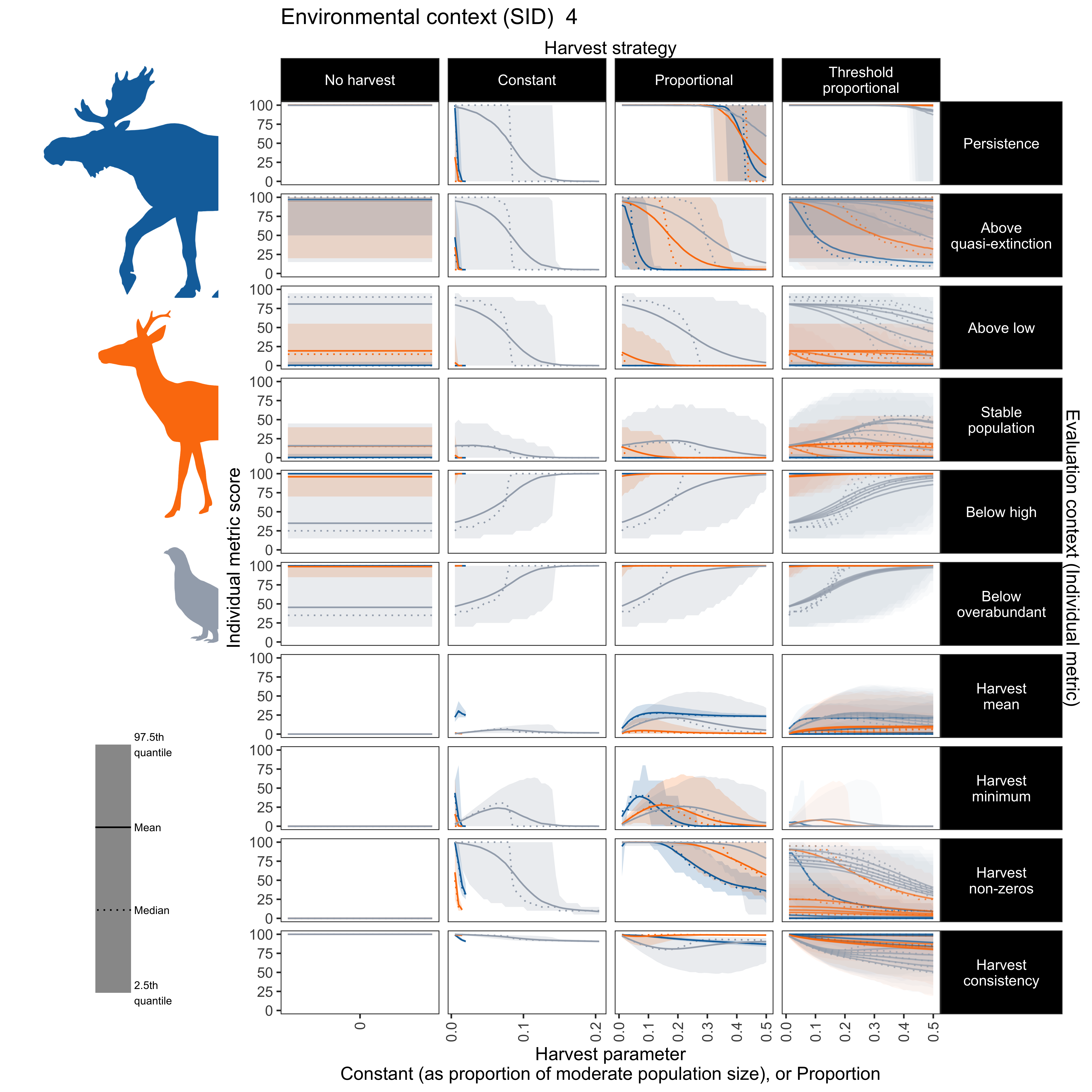


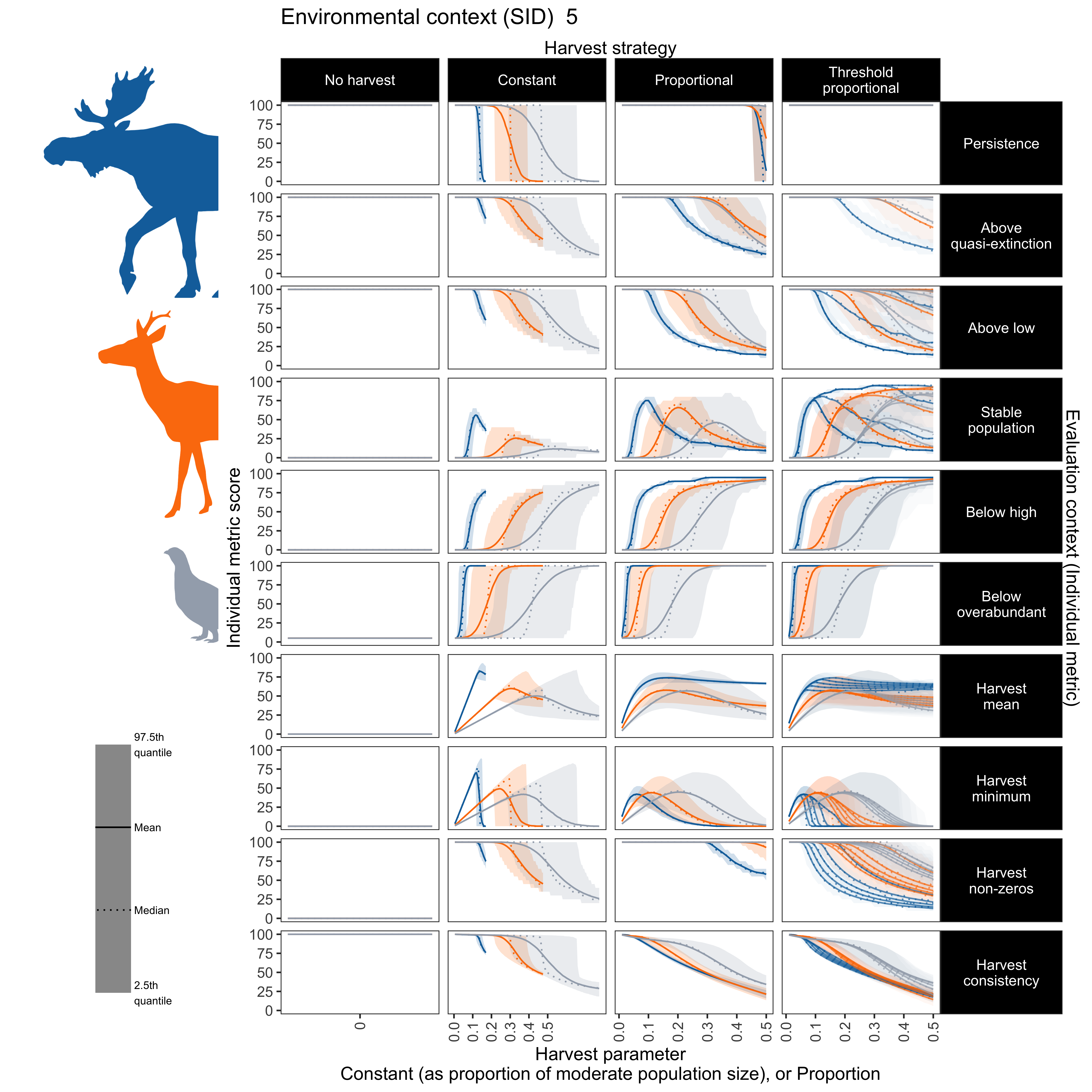


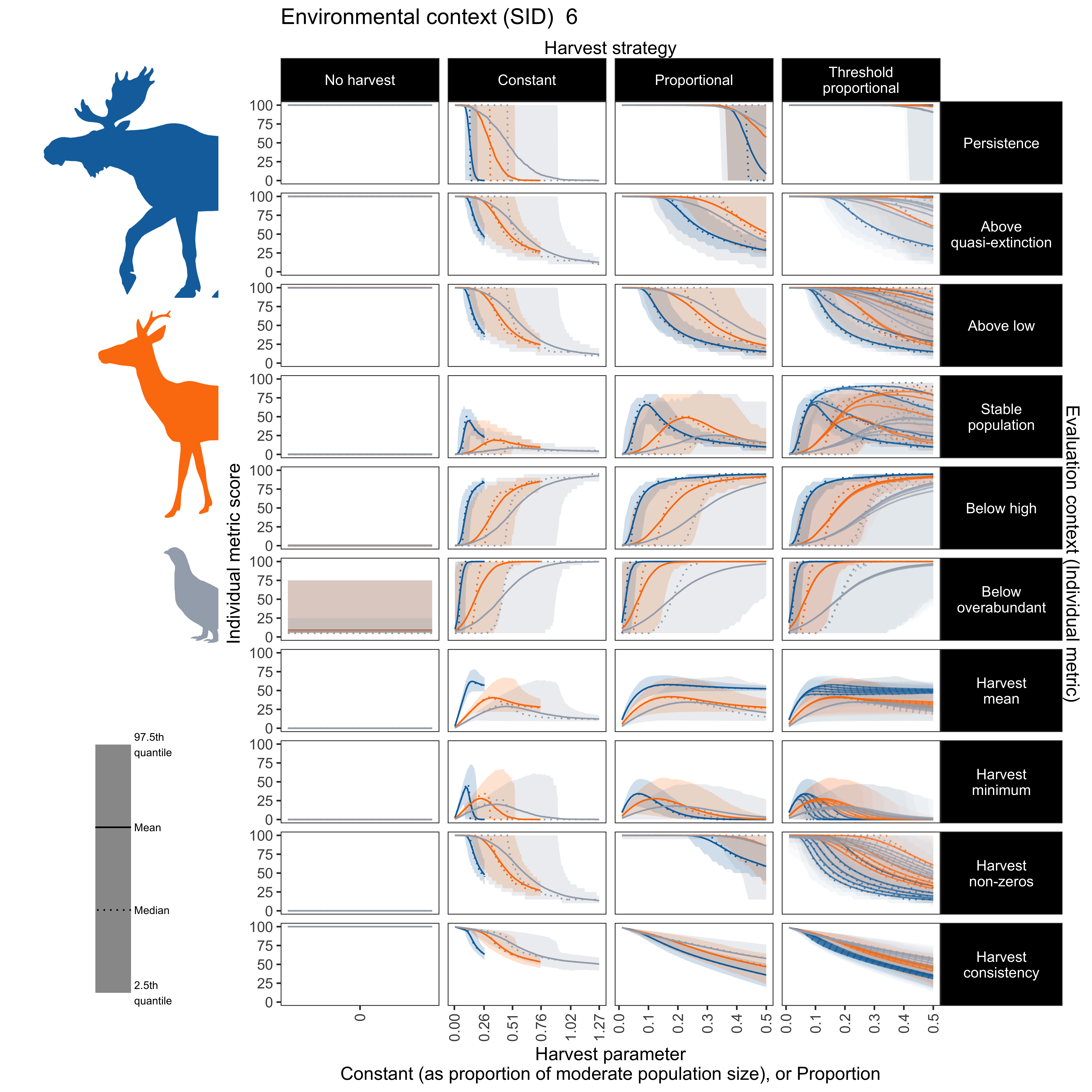


### Figure S2.1.2 Composite metrics, with variability

Expected (mean) scores, median scores, and the 95% Credible Interval for composite metrics for each species (colour), across the simulated harvest parameters (x-axis) for each harvest strategy (panel columns), and each evaluation context (i.e. composite set; panel rows). The threshold-proportional strategy is represented by 5 threshold values evenly sampling across the range of thresholds tested. Separate sets are given for each environmental context scenario (SID), as indicated by the set titles.


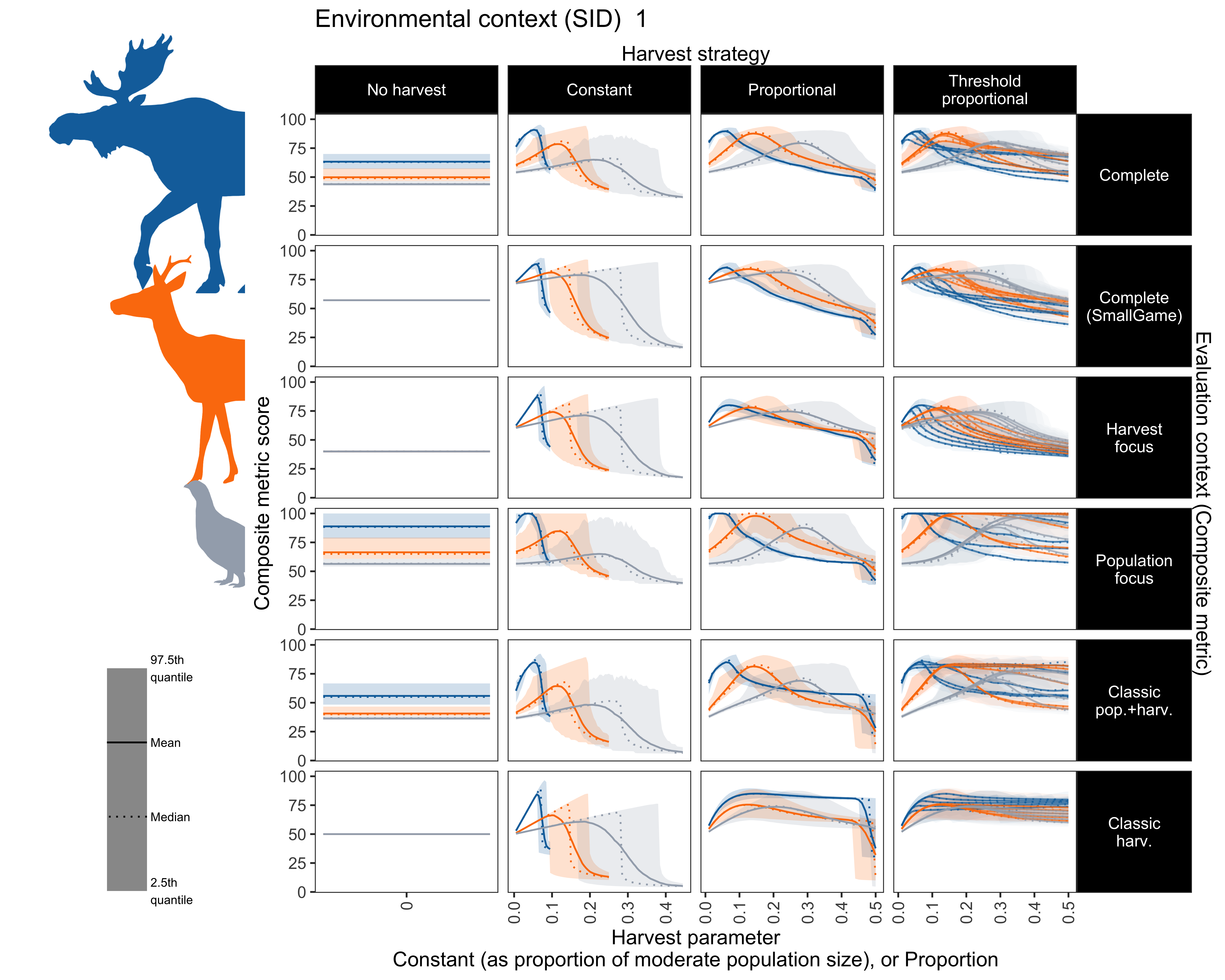


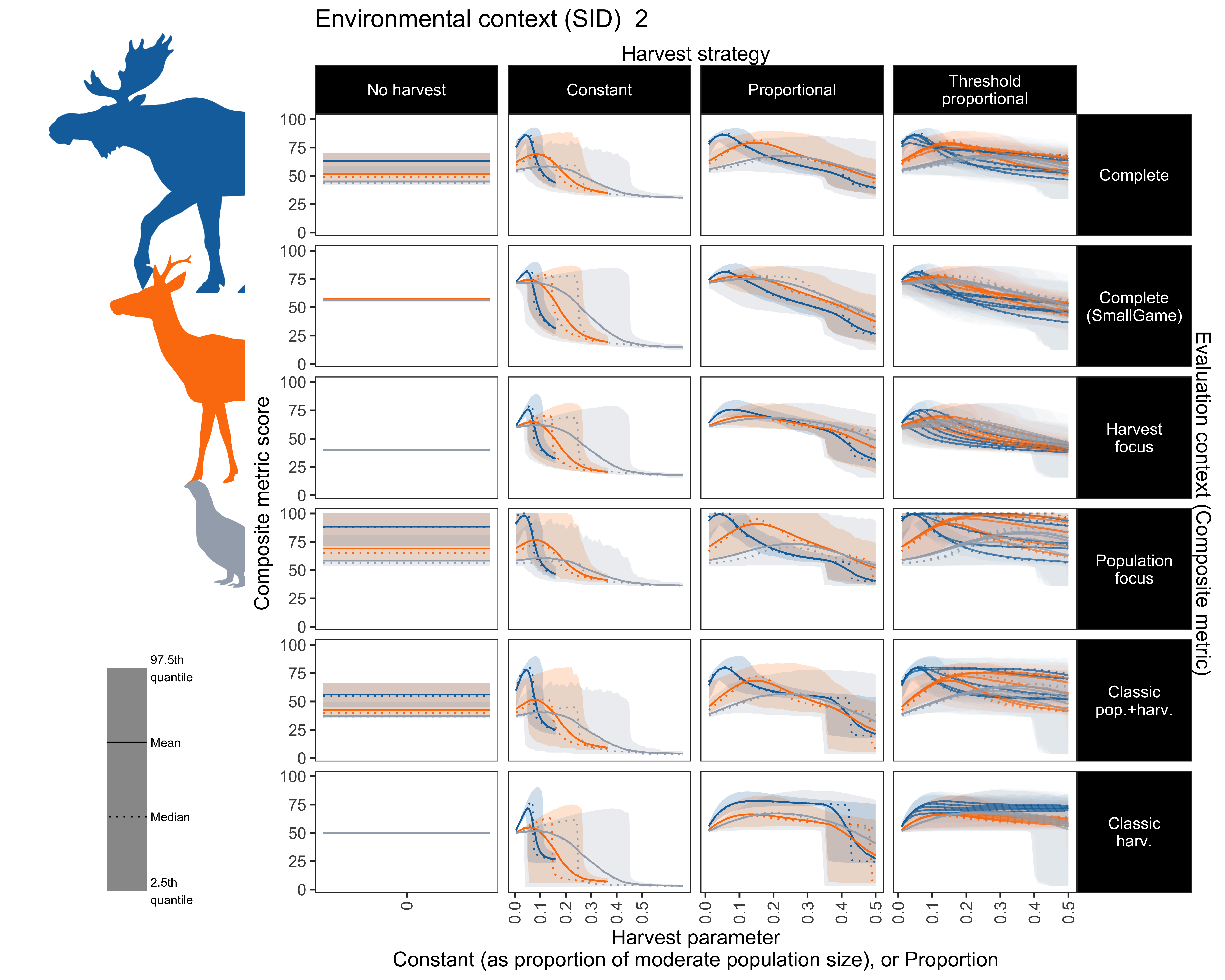


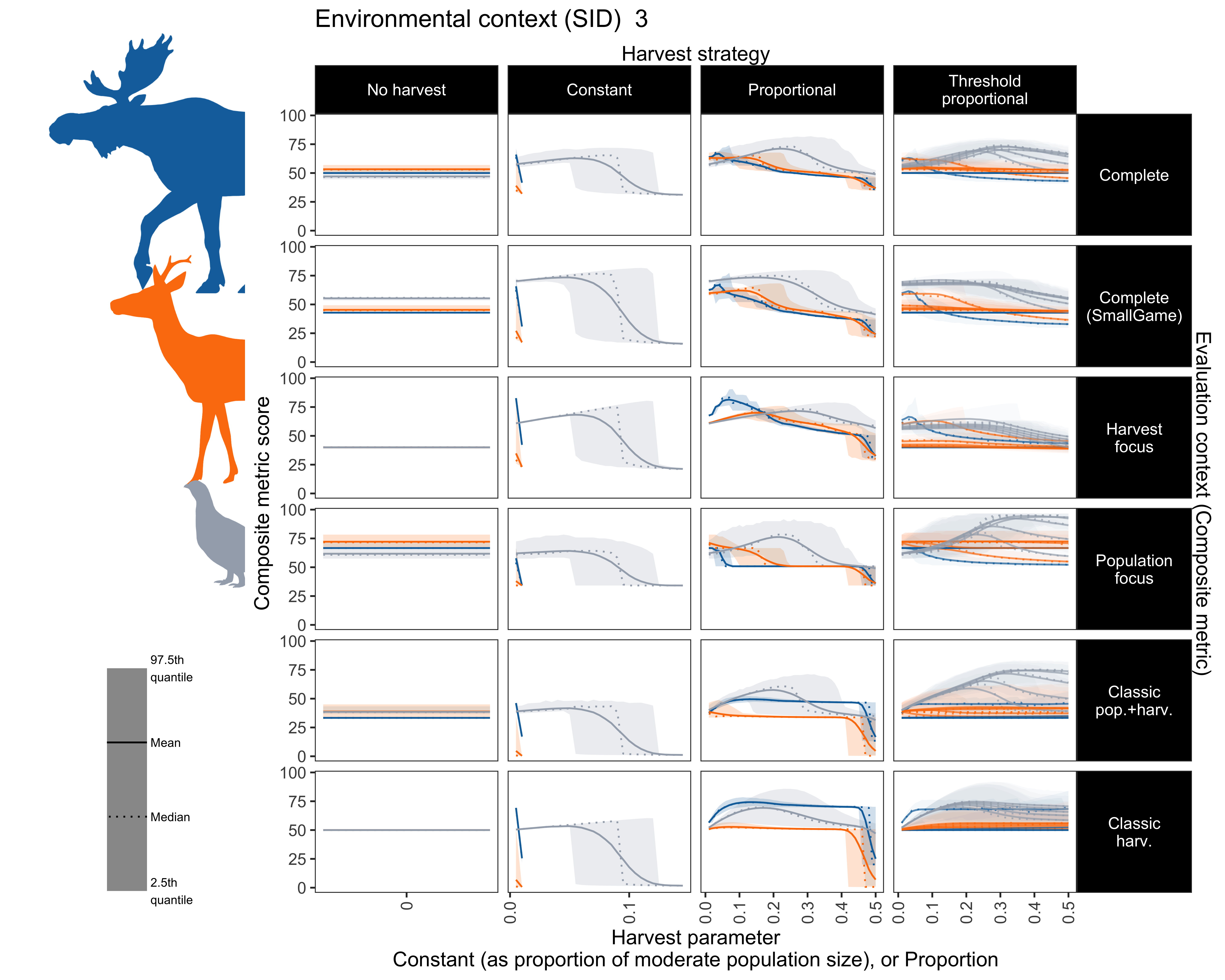


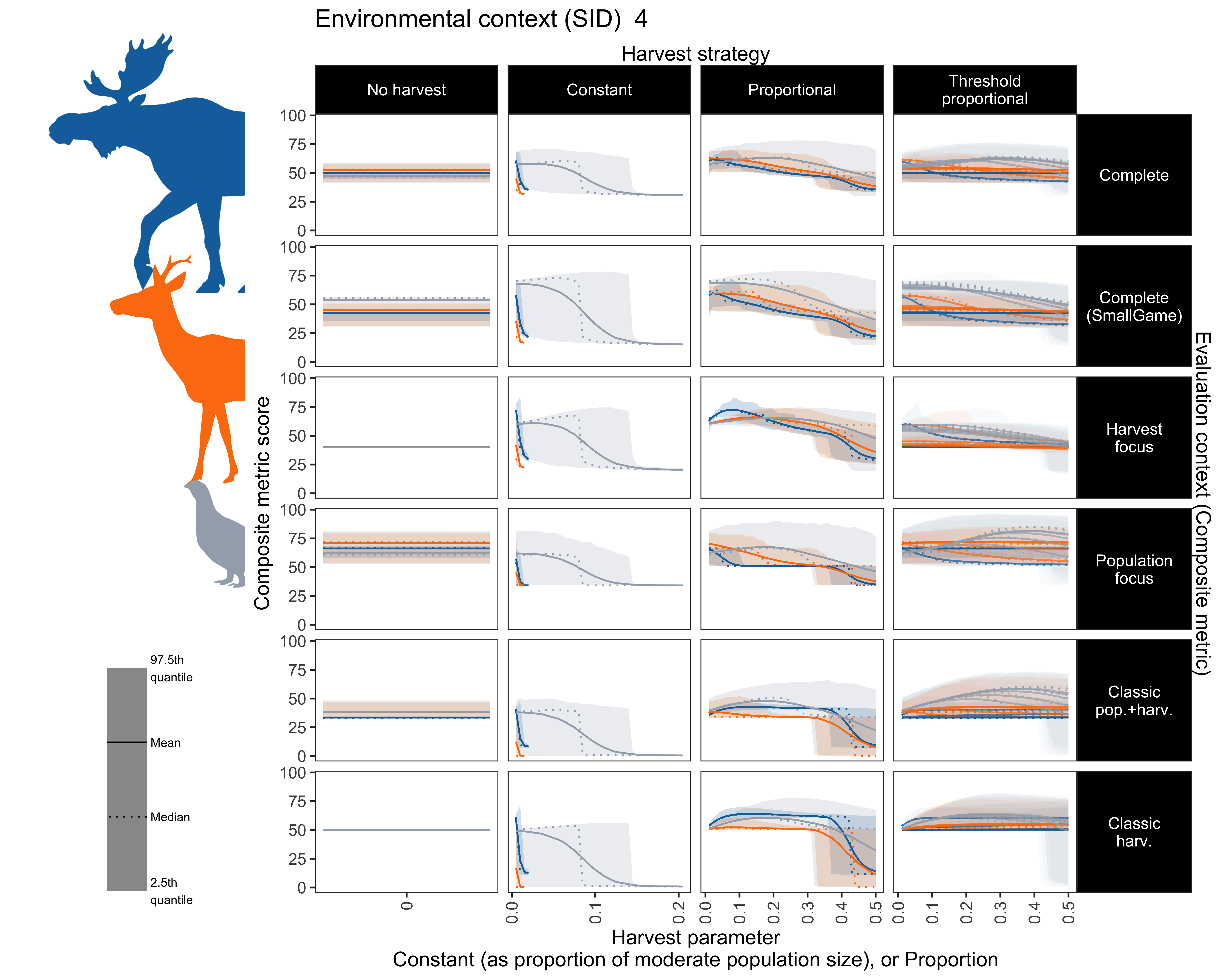


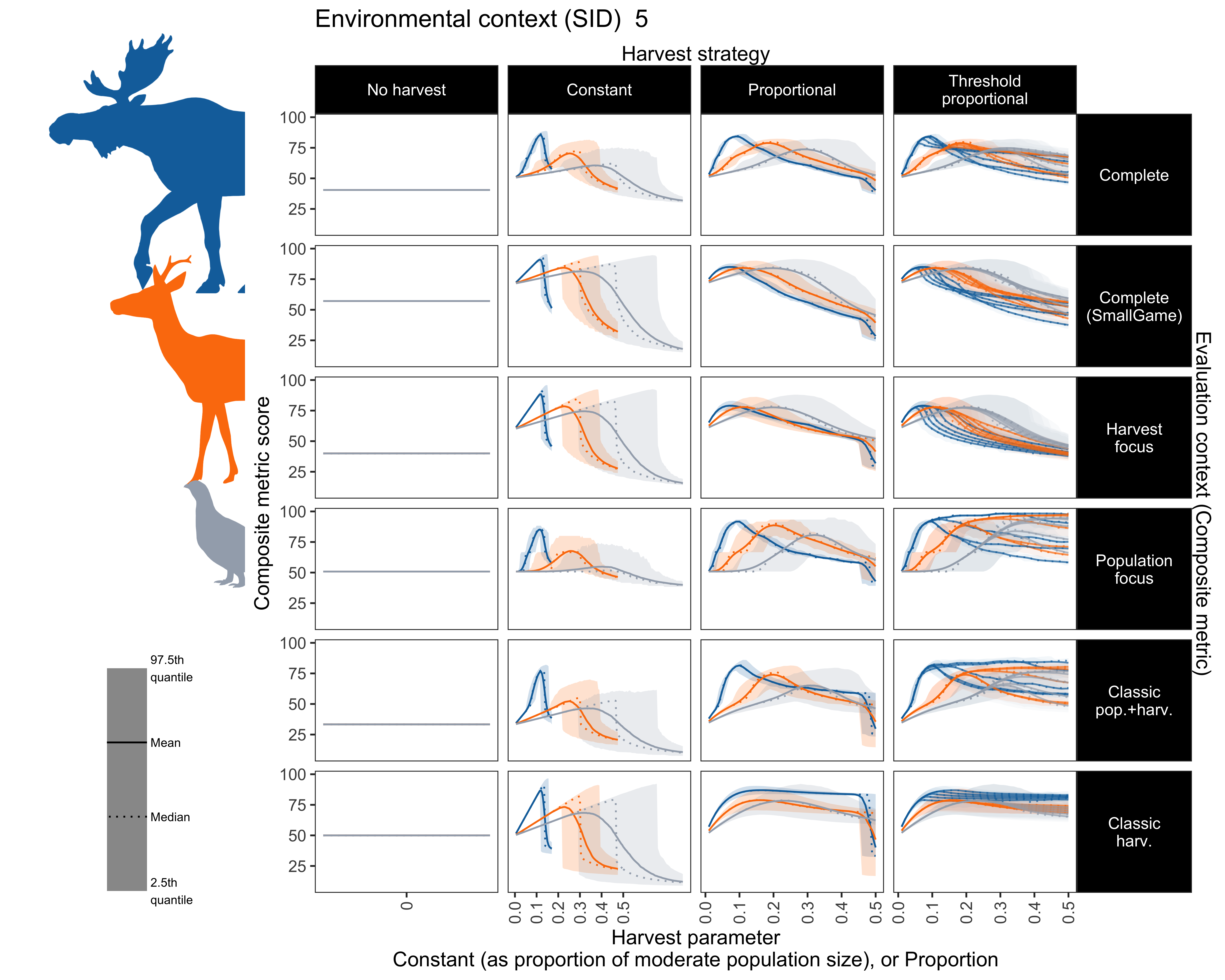


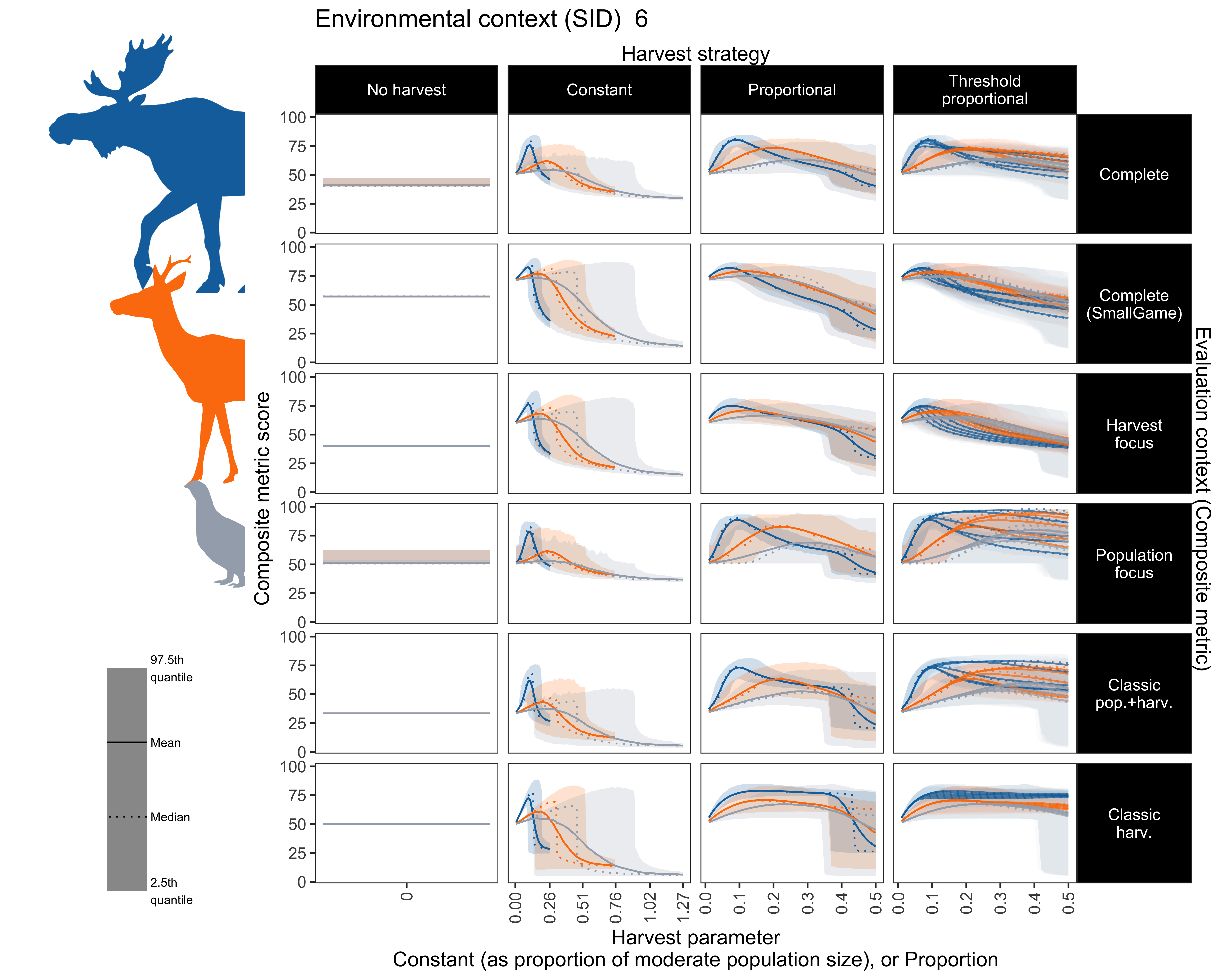


## S2.2 Optimal harvest levels for environmental and evaluation contexts within each harvest strategy

Optimal harvest levels are those which provide the highest score for the respective metric, in each scenario.

### Figure S2.2.1 Optimal harvest levels for constant harvests

Optimal harvest levels for constant harvest strategies. Figures show the parameter levels, i.e. constant harvest amounts (x-axis), that result in top scores (coloured areas with black outline) or within 5% of this top score (coloured areas), for each species (panel columns), evaluation context (composite metric, panel rows), environmental context (SID; y-axis).


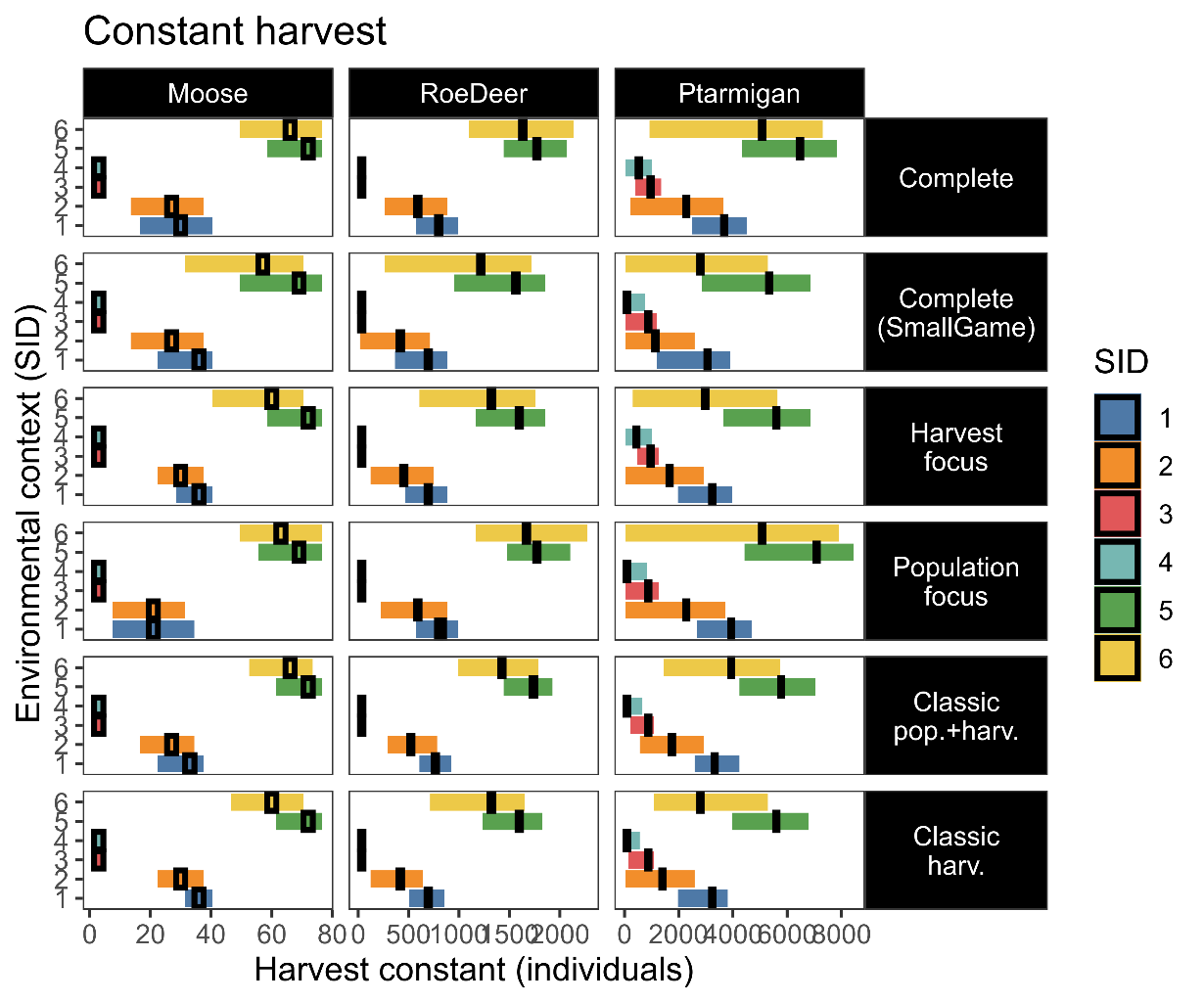


### Figure S2.2.2 Optimal harvest levels for proportional harvests

Optimal harvest levels for proportional harvest strategies. Figures show the parameter levels, i.e. the harvest proportion (x-axis), that result in top scores (coloured areas with black outline) or within 5% of this top score (coloured areas), for each species (panel columns), evaluation context (composite metric, panel rows), environmental context (SID; y-axis).


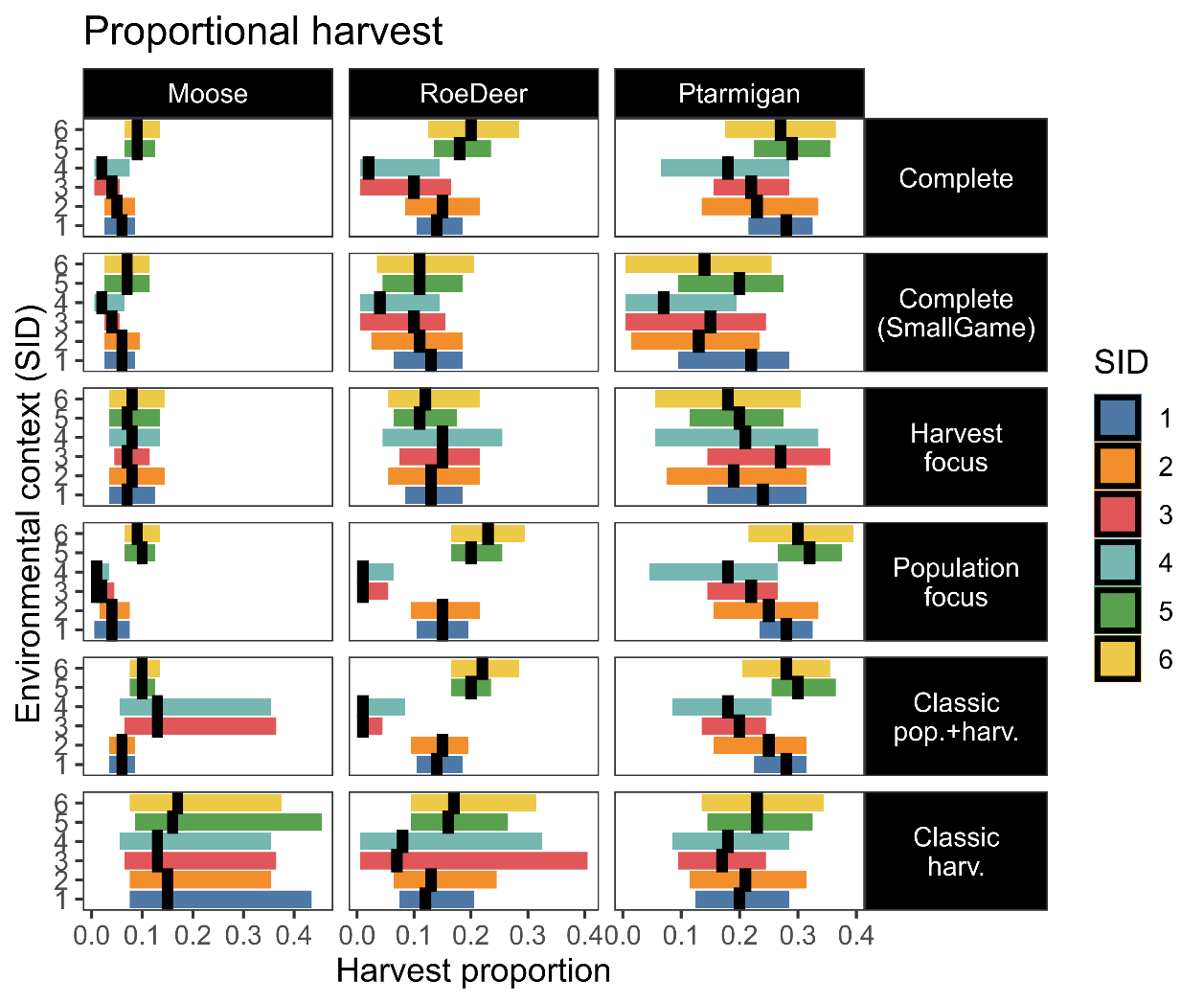


### Figure S2.2.3 Optimal harvest levels for threshold proportional harvests, under each evaluation context

Optimal harvest levels for threshold proportional harvest strategies. Figures show the parameter levels (x-axis showing threshold, and y-axis showing proportion) that result in top scores (coloured areas with black outline) or within 5% of this top score (coloured areas), for each species (panel columns), environmental context (SID; panel rows), and evaluation context (panel sets, indicated in the row facet labels).


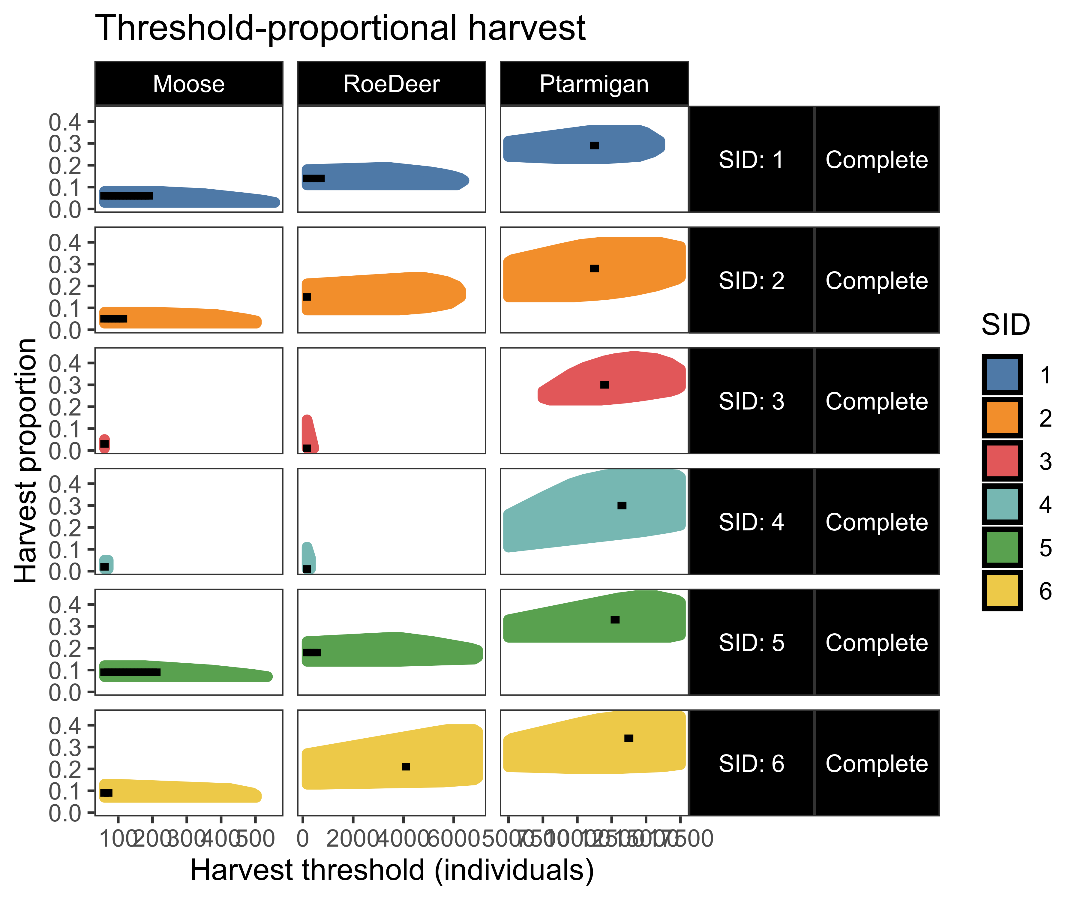


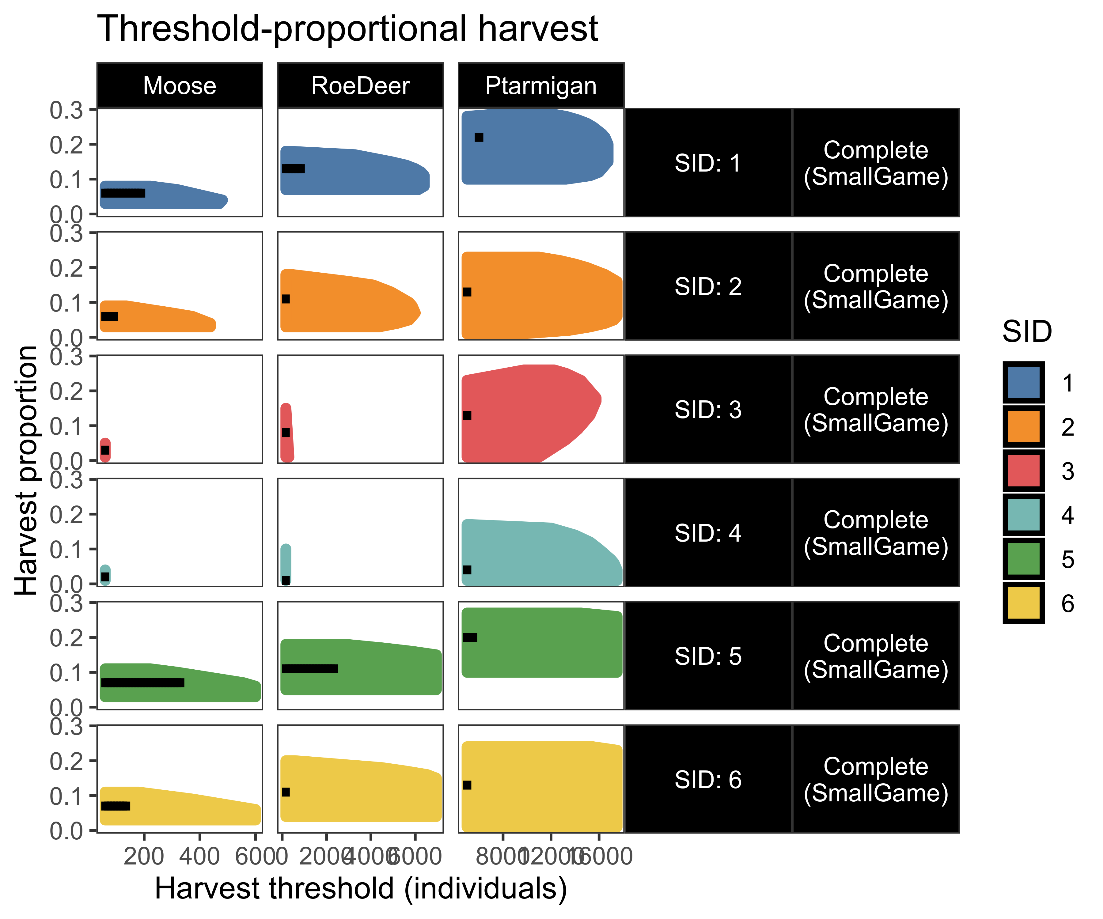


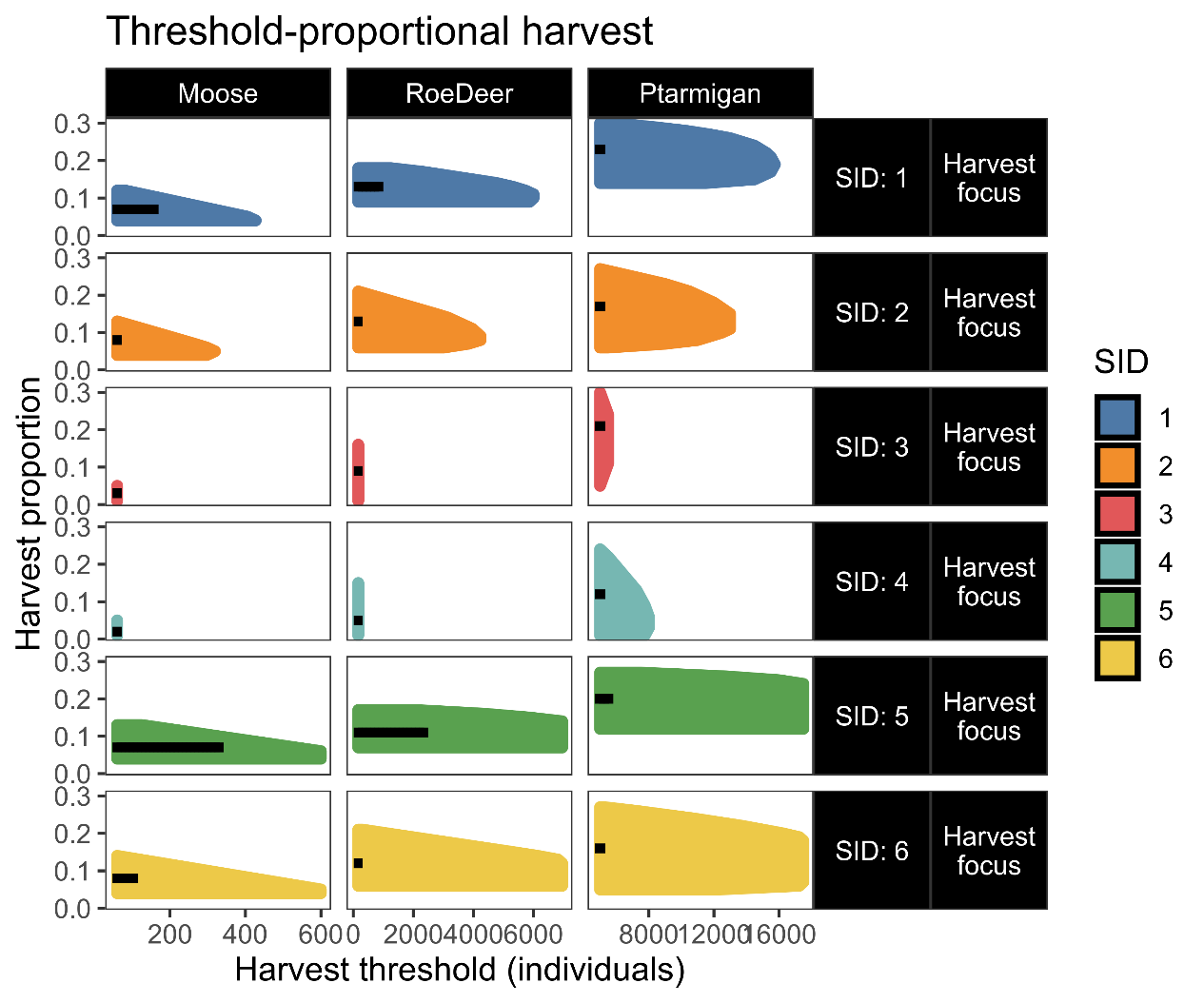


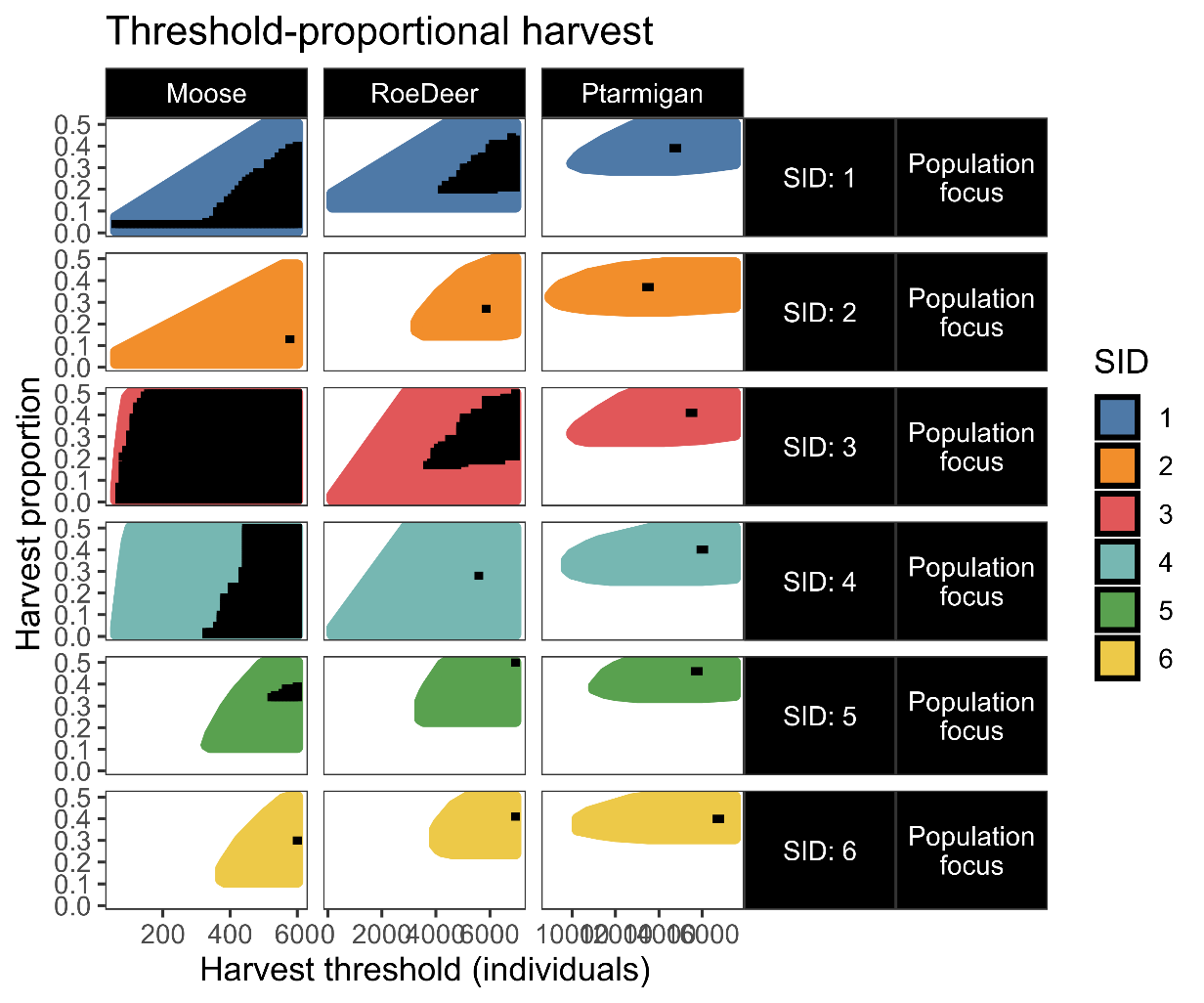


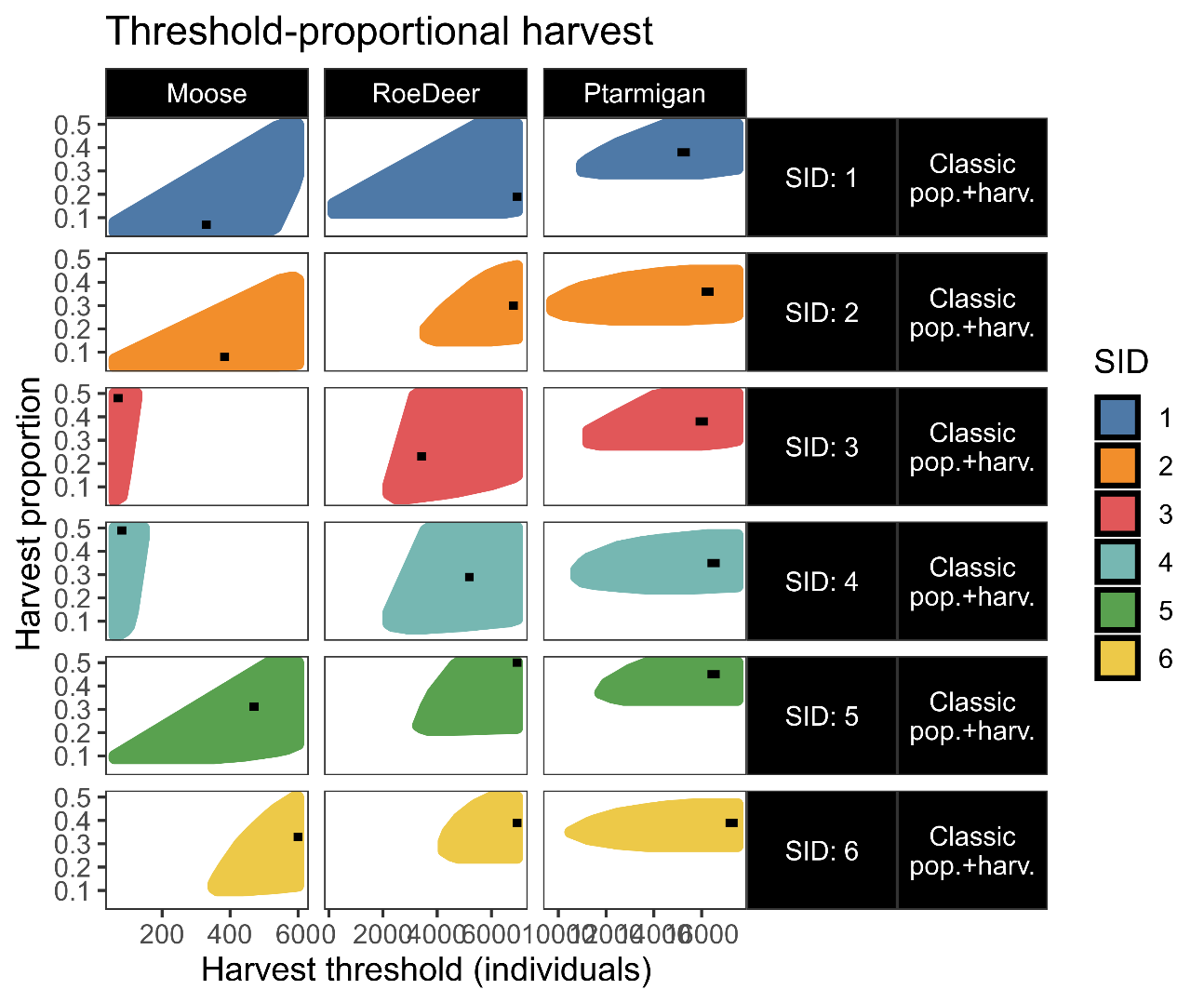

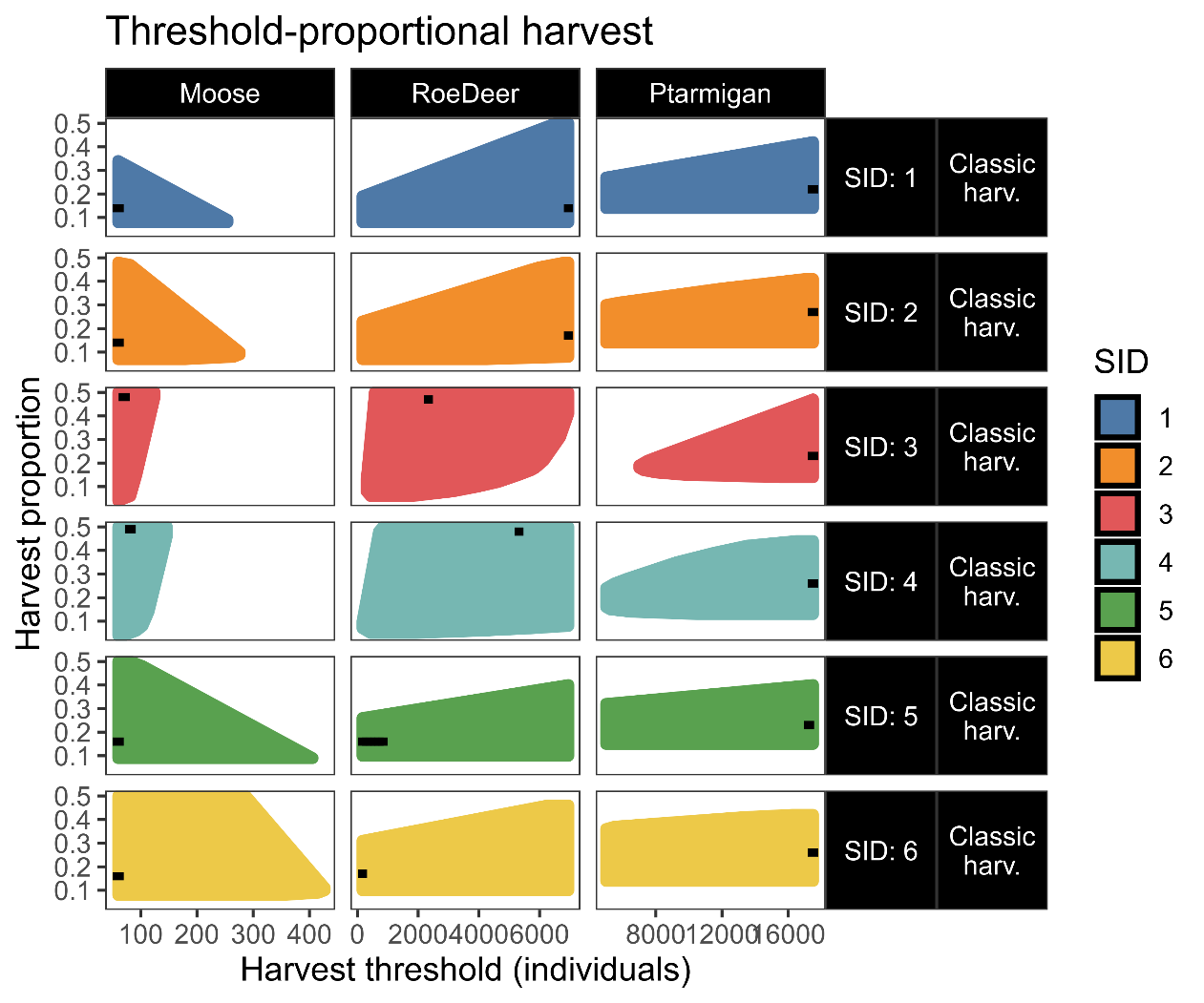


## S2.3 Optimal harvest parameter pairwise contrasts

Pairwise contrasts show the distribution of differences in pairs where the only factor that differs is denoted by the contrast stated. These contrasts therefore isolate the difference due solely to the identified factor contrasts.

### Figure S2.3.1: Pairwise differences in the optimal constant amount (constant harvest strategy)

Differences in optimal constant amounts under a constant harvest strategy (x-axis) due to differences in environmental and evaluation factors (y-axis), with all other factors held at equivalent levels for each pairwise contrast. Contrasts are given change in optimal constant amount for the left-hand level vs. the right-hand level, for example, the optimal constant harvest amount for moose is typically lower than that for roe deer, all other factors equivalent. Violins show the data distributions, with the colour indicating the median. Boxplots show the median, the first and third quartiles, and the whiskers extend to the smallest or largest value no further than 1.5 times the inter-quartile range from the hinge, with outliers plotted as points. Proportions of the observations below or above zero difference are given on the left and right grey panels respectively (and may not sum to one if some cases do not differ).
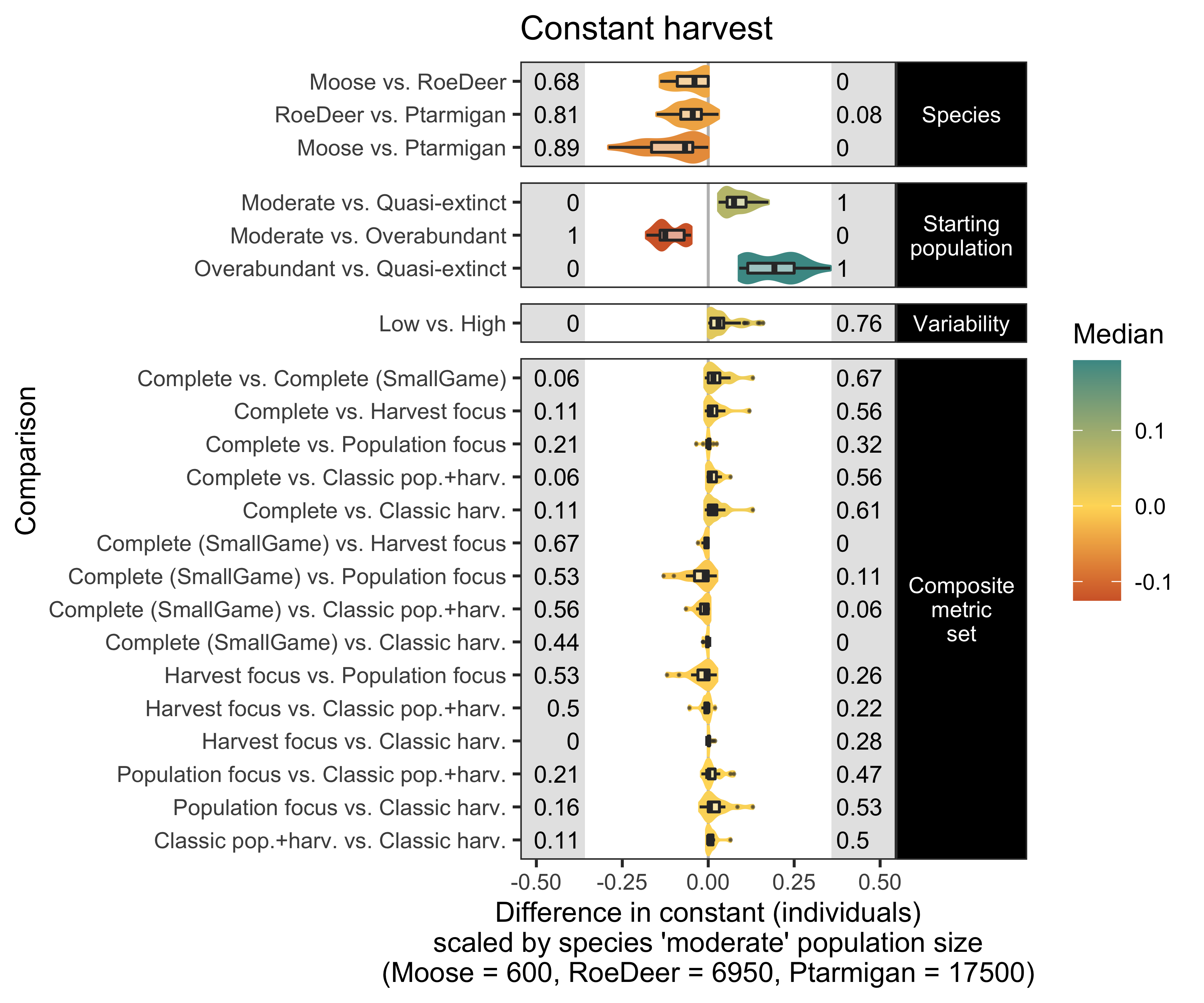


### Figure S2.3.2: Pairwise differences in optimal proportion (proportional harvest strategy)

Differences in optimal proportion under a proportional harvest strategy (x-axis) due to differences in environmental and evaluation factors (y-axis), with all other factors held at equivalent levels for each pairwise contrast. Contrasts are given change in optimal proportion for the left-hand level vs. the right-hand level, for example, the proportion harvested for moose is always lower than that for ptarmigan, all other factors equivalent. Violins show the data distributions, with the colour indicating the median. Boxplots show the median, the first and third quartiles, and the whiskers extend to the smallest or largest value no further than 1.5 times the inter-quartile range from the hinge, with outliers plotted as points. Proportions of the observations below or above zero difference are given on the left and right grey panels respectively (and may not sum to one if some cases do not differ).


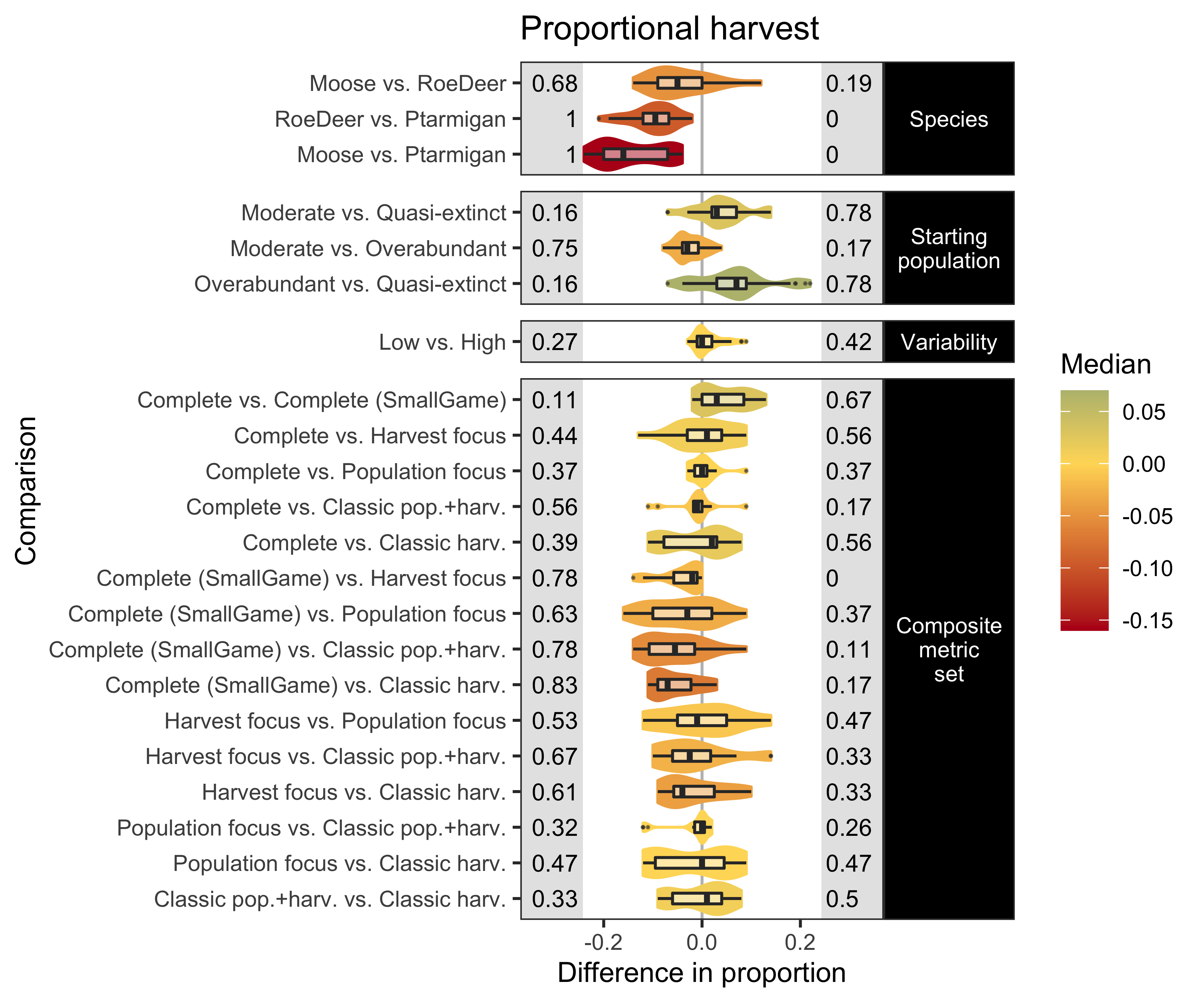


### Figure S2.3.3: Pairwise differences in optimal proportion (threshold-proportional harvest strategy)

Differences in optimal proportion under a threshold-proportional harvest strategy (x-axis) due to differences in environmental and evaluation factors (y-axis), with all other factors (including the threshold) held at equivalent levels for each pairwise contrast. Contrasts are given change in optimal proportion for the left-hand level vs. the right-hand level, for example, the optimal proportion for moose is typically lower than that for ptarmigan, all other factors equivalent. Violins show the data distributions, with the colour indicating the median. Boxplots show the median, the first and third quartiles, and the whiskers extend to the smallest or largest value no further than 1.5 times the inter-quartile range from the hinge, with outliers plotted as points. Proportions of the observations below or above zero difference are given on the left and right grey panels respectively (and may not sum to one if some cases do not differ).


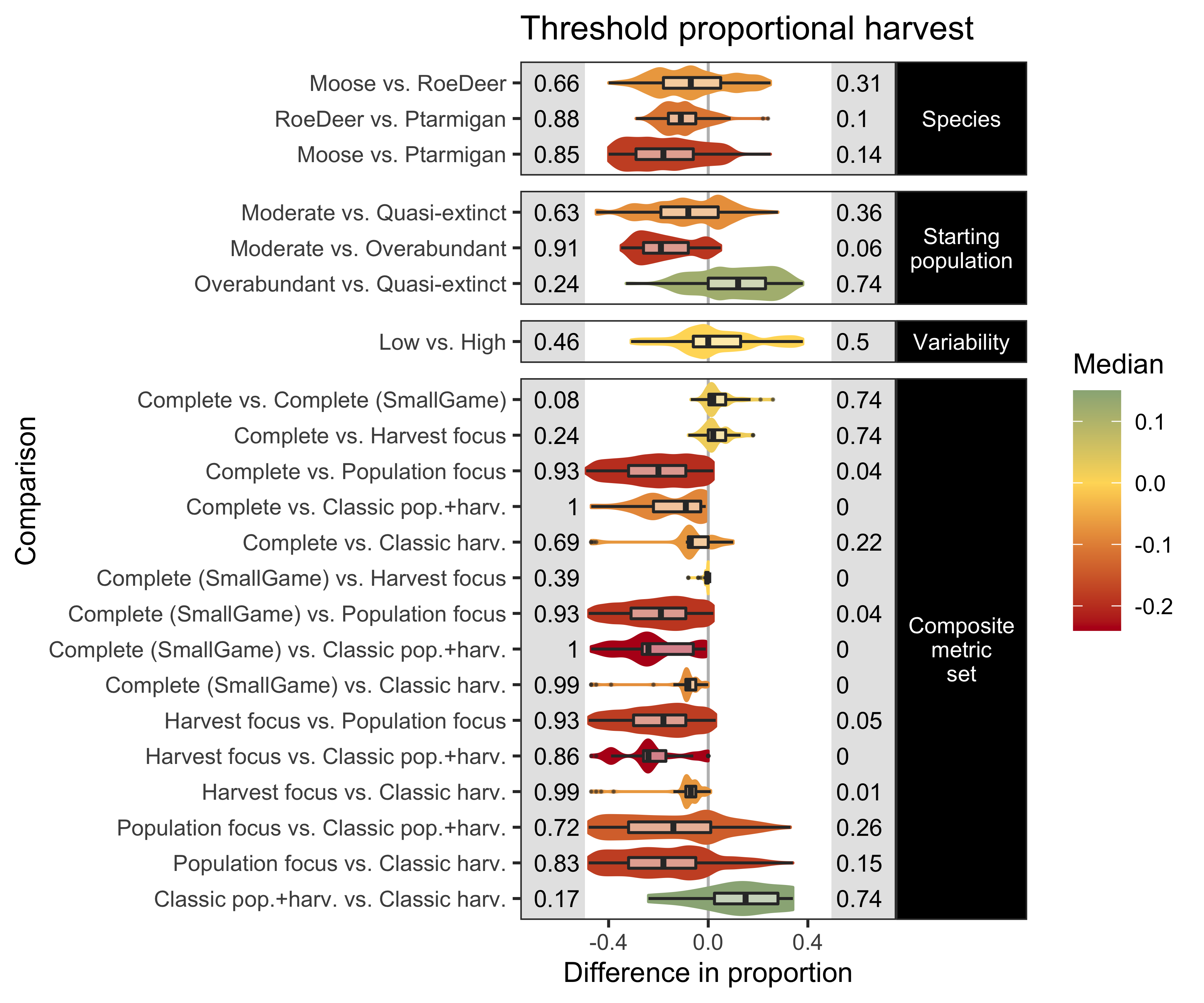


### Figure S2.3.4: Pairwise differences in optimal threshold (threshold-proportional harvest strategy)

Differences in optimal threshold under a threshold-proportional harvest strategy (x-axis) due to differences in environmental and evaluation factors (y-axis), with all other factors (including the proportion harvested) held at equivalent levels for each pairwise contrast. Contrasts are given change in optimal proportion for the left-hand level vs. the right-hand level, for example, the optimal threshold for moose is typically lower than that for ptarmigan, all other factors equivalent. Violins show the data distributions, with the colour indicating the median. Boxplots show the median, the first and third quartiles, and the whiskers extend to the smallest or largest value no further than 1.5 times the inter-quartile range from the hinge, with outliers plotted as points. Proportions of the observations below or above zero difference are given on the left and right grey panels respectively (and may not sum to one if some cases do not differ).


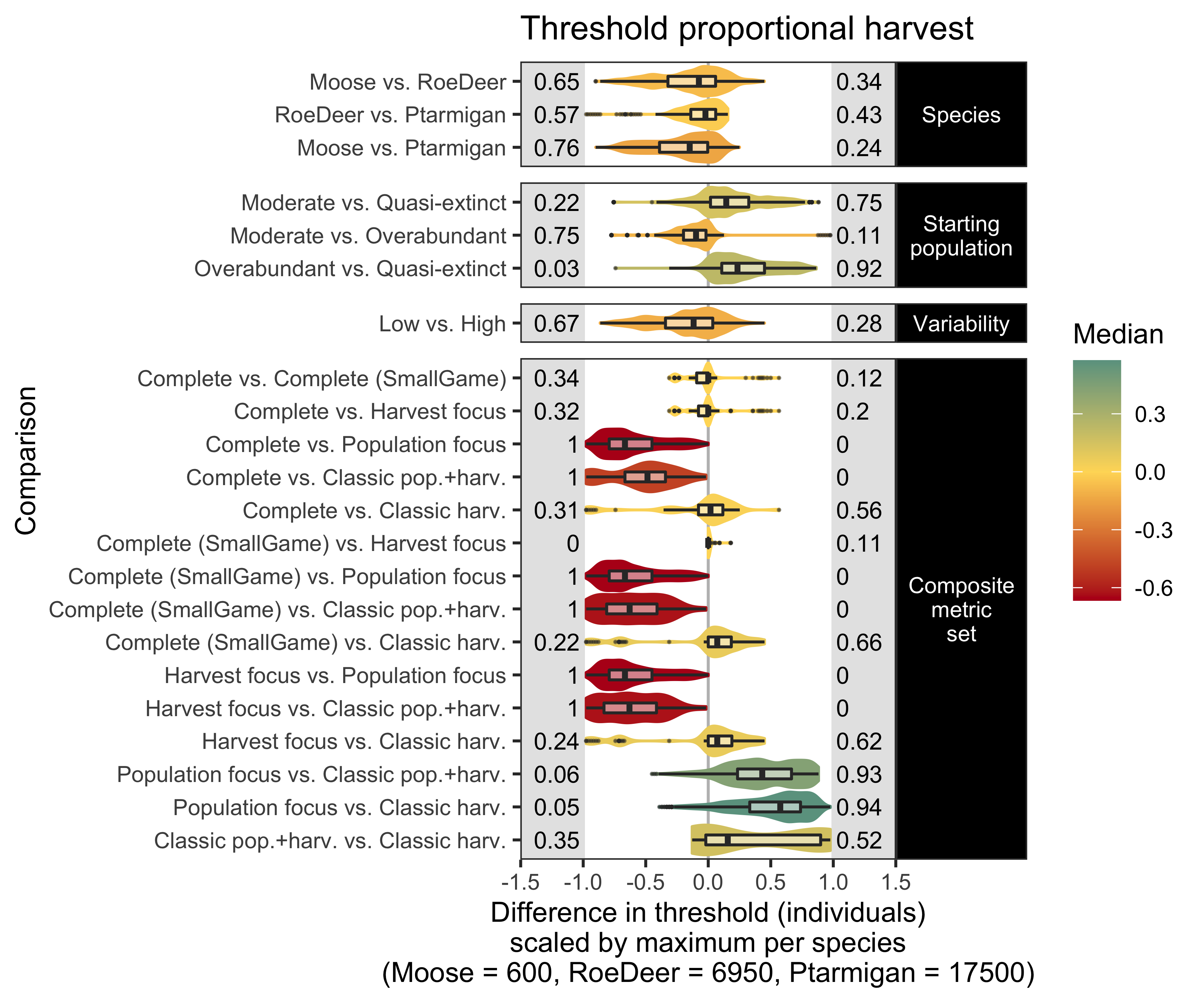


## S2.4 Pairwise factor contrast for optimal harvest strategies

Differences in optimal harvest strategies due to differences in environmental and evaluation factors (y-axis), with all other factors held at equivalent levels for each pairwise contrast. Contrasts are given as relative frequency (x-axis) of the type of change (colour) for the left-hand level vs. the right-hand level. For example, the optimal harvest strategy for moose is often a simpler strategy than for ptarmigan, all other factors equivalent. Harvest strategies are ranked from simple to complex as follows: no harvest, constant harvest, proportional harvest, threshold proportional harvest. Change categories are denoted as “simpler” when the optimal strategy or strategies all become simpler, e.g. when changing from proportional to constant, and “more complex” when the strategies become more complex. As there are often multiple optimal strategies in any particular case, change categories of “+ simpler” or “ – simpler” denote when a simpler harvest strategy is (respectively) added or removed from the optimal strategy set for a particular case. Likewise, “+ more complex” and “ – more complex” represent when a more complex strategy is added or removed from a set.


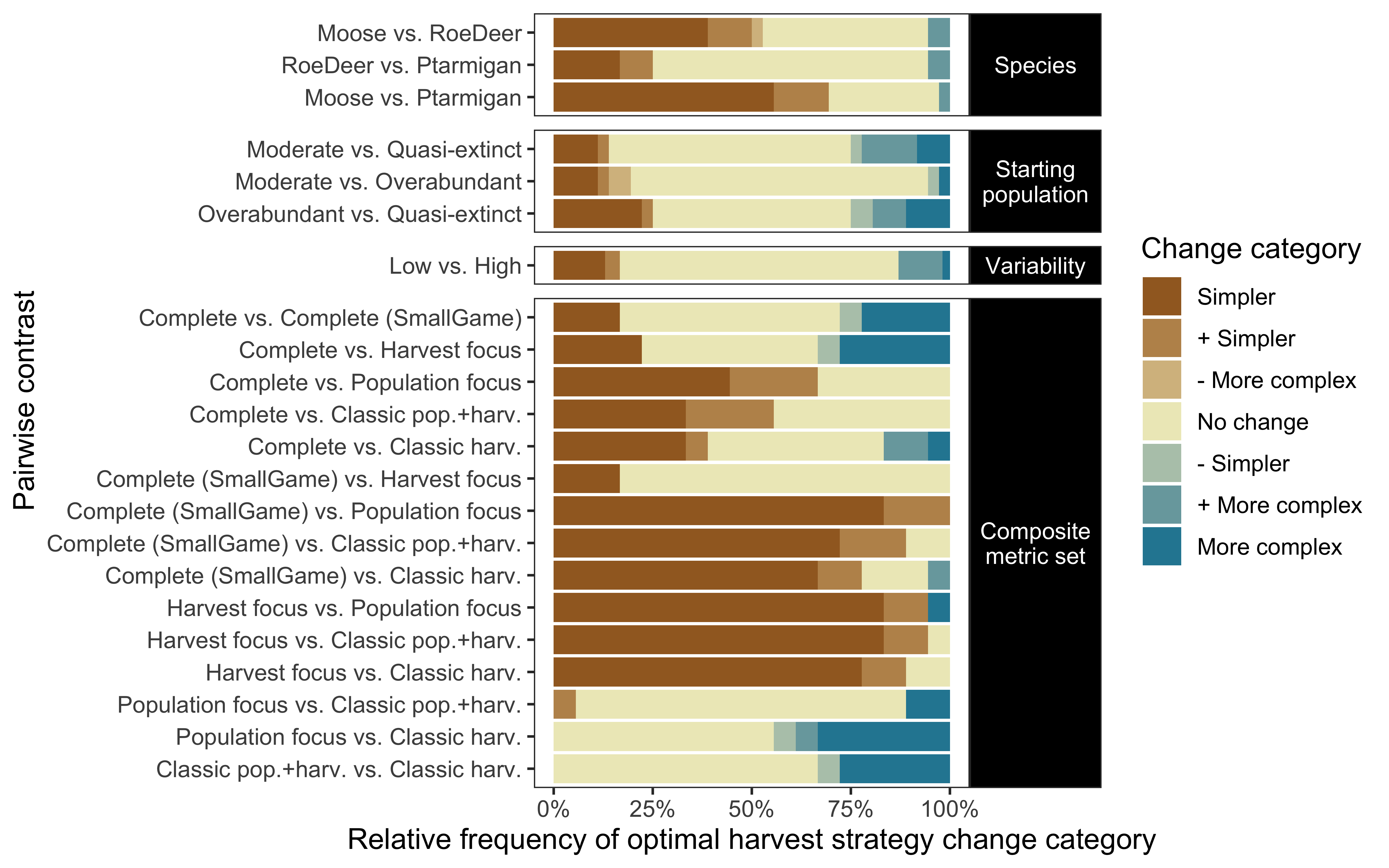

Supplement: S2 Appendix — (DOCX) [file pone.0260159.s002.docx]
