# Supplementary material for: Heuristics for the sustainable harvest of wildlife in stochastic social-ecological systems
Source: PLoS One. 2021 Nov 19;16(11):e0260159. doi: 10.1371/journal.pone.0260159 (PMC8604319; doi:10.1371/journal.pone.0260159)
Supplement: Attachment — Submitted filename: Comments reply 20210922.docx [file pone.0260159.s003.docx]

Response to reviewers

**PONE-D-21-14753
Heuristics for the sustainable harvest of wildlife in stochastic social-ecological systems**

[Comments, numbered, aligned, in blue]

[Responses, inset, in green, with text from manuscript in underline]

# Editorial comments

[Comment E1] Thank you for submitting your manuscript to PLOS ONE. After careful consideration, we feel that it has merit but does not fully meet PLOS ONE’s publication criteria as it currently stands. Therefore, we invite you to submit a revised version of the manuscript that addresses the points raised during the review process.

Please consider the comments from both Reviewers. In particular, please address the suggestion to define sustainability from Reviewer 1. Given that this term is ill-defined (line 48), how do we know that this paper captures it, as suggested by the title? Also, please discuss alternative ways to combine information across performance metrics (e.g., MinMax, or minimax), as suggested by Reviewer 2.

[Reply E1] Thank you for the opportunity to revise our manuscript for PLOS ONE, and for the many useful comments to guide this. We have revised the manuscript to more clearly provide our working definition of sustainability (see reply to comment R1.1 and R1.2) and now include a discussion of alternative performance metrics (see reply to comment R2.2, and address the other comments individually below.

[Comment E2] Also, please consider the following comments and editorial suggestions.

1. Much of the focus of MSE in fisheries has moved away from identifying single best harvest strategies to identifying those that “satisfice” stakeholder criteria to avoid over-reliance on models assumptions that often do not reflect reality (e.g., [Harford and Carruthers 2017](https://www.sciencedirect.com/science/article/pii/S0165783617300310),). This paper diverges from that approach because of its focus on identifying heuristics for data-limited contexts. I suggest mentioning how and why this paper diverges from the current paradigm in fisheries MSE in the Introduction.

[Reply E2] We have included mention of this aspect in the introduction “…even in the best studied cases, important elements of the social-ecological system remain uncertain or contested [24–28], and there is a trend away from defining single ‘best’ harvest strategies, in order to avoid over-reliance on models with assumptions that may not hold in reality [Harford and Curruthers 2017].” [line 59]. This is followed by the paragraph introducing heuristics, in which we provide three key arguments why we believe heuristics are useful – not just for data-poor management scenarios (where heuristics are aimed at providing management recommendations robust to uncertainties), but also in knowledge-rich scenarios (for guiding sensitivity analyses, and context comparability).

[Comment E3]

2. Did you consider ecological objectives. Are these species important prey or predators? I suggest describing how the performance metrics (evaluation metrics) chosen are a subset of a much larger set of possible metrics, including those related to income/jobs from harvest which are not directly related to number harvested and depend on the management strategy.

[Reply E3] We now introduce the modeled species (“moose, *Alces alces,* roe deer, *Capreolus capreolus,* and willow ptarmigan, *Lagopus lagopus”* [line 101]) earlier, in the introduction. We emphasise that these are common prey species, as we describe more fully in the methods (in the section “Environmental context and decision variable parameters” [line 149]). We now also clarify in the discussion, that “We included prey species only, and do not explicitly consider biotic interactions such as their natural predators (these are assumed as captured within the variable demographic rates).” [line 491]

Regarding the evaluation metrics, we have revised the manuscript to introduce this with more detail in the introduction, specifying that “we evaluate sustainability over 10 evaluation metrics reflecting volumes and variability of both population and harvest, combined into 6 stakeholder perspectives relevant for terrestrial contexts” [line 105]. The methods section explains these further – defining the objectives in Table 1 – and we now include here the caveat that: “While we aimed to include as broad a range of sustainability metrics as possible, these may not be adequately representative of objectives that are not rooted in volumes or variability of either harvest or population sizes” [line 191].

[Comment E4]

3. Why is performance poor on all metrics with no harvest? (even population persistence scores)? (Fig. 3)

[Reply E4] Results for *Individual metrics* are given in the supplementary S2.1. These clearly show population persistence scores are at their highest with no harvest.

As described in the figure legend, Figure 3 shows *composite scores*. Composite scores are functions of multiple individual metrics, as described in the methods. Therefore, to have a high score in a composite metric, all the contributing scores need to also be high.

Low scores in a composite metric may be due to two reasons. First, conflicts between individual metrics mean that both are not achievable simultaneously – for example, minimizing the chance of under-abundance (for example, via not harvesting) often comes at the risk of increased over-abundance. Second, sometimes high individual metrics are simply not biologically possible – for example, if populations start very small, with a small growth parameter (lambda), these are unlikely to recover to reasonable sizes within the timeframe considered.

The majority of the composite metrics (except for ‘population focus’) include some measure of harvest. Thus, in the ‘no harvest’ strategy, the harvest metrics will be zero, resulting in low scores for these strategies. Meanwhile, the ‘population focus’ metric forgoes the harvest metrics, but includes consideration of both under-abundance and over-abundance. Given our parameters (specifically, a positive lambda for the logistic growth equation), without harvest our populations typically become over-abundant. The exception is the moose, which – provided it starts with moderate abundance (SID 1-2) – does not readily become over-abundant. However, if it starts as a very small population (SID 3-4), the lambda does not allow a fast enough increase to recover the population to reasonable levels, even without harvest. Starting in an overabundant population (SID 3-4), clearly with no harvest to reduce this, low composite metrics scores are due to overabundance.

[Comment E5]

4. Why is performance poor for moderate, but not high and low environmental variability for Moose (and some extent Roe Deer)? (Fig. 3)

[Reply E5] Environmental variability includes two levels: low (SID 1,3,5) and high (SID 2,4,6). The starting population size includes moderate (SID 1-2), low (SID 3-4) and high (SID 5-6). As such, we are not sure exactly what this comment refers to. As we summarise in the results (including figures 4 and 5), moderate starting population sizes typically do better than high starting sizes, and both of these better than low starting population sizes. The very rare exceptions (for example Moose, in Constant or Threshold Proportional harvest, under a Classic Harvest composite metric) are due to the higher harvests being feasible with an overabundant starting population, compared with the moderate starting population. Note, this only occurs with low variability scenarios. Low variability scenarios almost always perform better than high variability scenarios – and we explain the rare exceptions in the discussion (these are likely due to stochastic resonance).

[Comment E6]

5. Results depend on the caveats and assumptions of the model as partly explained in S1. Given that these assumptions are largely untested, they should be brought forward to the Discussion. For example, how realistic is the performance metric on extinction given limited knowledge about population dynamics at low abundances (i.e., allele effects).  Also, alternative distributions of population variability could be considered with longer tails to account for infrequent extreme events that strongly impact on performance (e.g., [Anderson et al 2017](https://www.pnas.org/content/114/12/3252) https://www.pnas.org/content/114/12/3252). This can be included as a caveat in S1.1, and brought forward to the main discussion as well. In addition, biases in observation errors and harvest implementation can significantly impact performance, yet are not included and can be highlighted in the Discussion.

[Reply E6] We have revised the manuscript to bring more of the caveats into the main text discussion. This now includes a section highlighting these: “Further developments of the model would be valuable to extend the results to more complex and realistic contexts, in particular to examine impacts of particular sources of uncertainty and variability (e.g. of population structuring during low abundances and infrequent extreme events [(Lacy 1993, lacy and Pollack 2020, Anderson et al 2017, Youngflesh and Lynch 2017)]), and directional biases (e.g. in monitoring, quota setting, and harvest [(Eriksen et al 2018, Bieg et al 2017)])” [line 496]. We also have revised the supplementary to include these further examples.

We agree that the performance metric based on extinction only is typically poorly representative of broader ecological concerns (e.g. ecosystem functionality), and also exposes populations to such risky and uncertain low-population dynamics. This is why we wanted to include a fuller range of evaluation metrics than are typically included in the harvest literature. However, we did not consider very rare species – these are unlikely to be harvested. Populations of our prey species may be locally rare, but then potentially not subject to such Allee effects due to immigration (which we also don’t consider). Indeed, a letter reply to Anderson et al 2007, by Youngflesh and Lynch (https://doi.org/10.1073/pnas.1713621114), point out that such movements are pivotal to consider in changes in abundance, so, for any inclusion into the model of e.g. Allee effects needs to also consider structured populations, resulting in a much more complex model.

As we highlight in the discussion already, our models are biologically simple, and we do this intentionally so that we can bring in more contextual variability and more objective complexity than prior work. With this added contextual and objective complexity, we saw no advantage to overloading what is already a challenging manuscript with further biological complexities. This might however be interesting for future studies.

[Comment E7]

6. Given the sensitivity of results to environmental variability, evaluation context, etc. and all the caveats of missing components/assumptions highlighted in S1 and by Reviewer 1, the conclusions and abstract should highlight the need for adaptive *and precautionary* management. This is especially true under climate change where future scenarios may not resemble historical conditions/trajectories or those simulated in the MSE.

[Reply E7] We have revised the abstract to better emphasise how our results support the use of adaptive strategies, by including “Despite not always being the optimal solution, overall adaptive harvest strategies resulted in the least value forgone, and are likely to give the best outcomes under future climatic variability and uncertainty.” [line 33]

We have also modified the discussion to mention how adaptive strategies are a key “precautionary approach [to management] under uncertainty and variability” [line 423], and further introduce this terminology in the discussion section dealing with slower life history species: “Overall, this supports adaptive harvest strategies (including proportional and/or thresholds) as a precautionary strategy, providing economic and ecological resilience of harvest under both scientific and environmental uncertainty, and particularly uncertainty in the face of directional threats such as climate change”. [line 484]

[Comment E7] Editorial suggestions

- The difference between single composite metrics and holistic metrics is not immediately clear in the abstract on first read. I suggest giving brief examples in the abstract to illuminate your point.

[Reply E7] We have modified this section to clarify: “Importantly, simple composite metrics popular in the theoretical literature (e.g. focusing on maximizing yield and population persistence only) often diverged from more holistic composite metrics that include a wider range of population and harvest objectives, and better reflect the trade-offs in real world applied contexts.” [line 28]

[Comment E8]

- Is 1000 MC trials enough for risk-based performance metrics, especially those that pertain to low frequency events like extinction?

[Reply E8] In general, we agree that 1000 MC trials would not be not enough for a simulation analysis. However, as we iterate over multiple harvest levels, this number should be seen as single observations across a gradient, rather than of an individual independent scenario.

[Comment E9]

- How were the individual sustainability metrics chosen? (based on previous experience with management of those 3 species in particular, review of the literature, etc.?)

[Reply E9] We now clarify in the S1.1 section on evaluation, that: “Evaluation metrics are designed to reflect different potential objectives and stakeholder concerns, and cover a number of socio-ecological (i.e. population-based) and harvest-based sustainability objectives (Table 1). These were gathered from both theoretical literature and empirical collations of harvest objectives stated in wildlife harvest policy documents (e.g. Artelle et al. 2018), and reduced to those feasible given the data generated by the model, without the need for additional subjective valuation metrics.” [S1 line 130]

[Comment E10]

- The “Complete” composite score weights all metrics equally, so is a function of the number of metrics on each category. It would be more transparent to label it as equal weighting to population and harvest. “Complete” may be mean different things to different stakeholders.

[Reply E10] We respectfully disagree with renaming this to ‘equal’. All composite metrics have equal weights over their respective included metrics, and feel that describing the composite score containing all possible metrics as ‘Complete’ is well defined in the context of our study – and given that it may mean different things to different stakeholders, we feel that readers will seek out the definition.

As a side, we originally included equal weighting of population, harvest, and persistence metric groups, but decided against this additional complexity. Such a weighting did not increase the importance of persistence as an objective in practice (as it is ‘required’ for most other objectives anyway), and only served to reduce the measurable differences between scenario outcomes (by reducing the more variable components of the scores).

[Comment E11]

- I suggest adding a description of the range of harvest parameters to the main text. It’s not clear how 289, 848 model runs are generated (line 213) from the text alone.

[Reply E11] We have modified the methods in the main text to clarify: “We searched across a wide range of constants, proportions, and thresholds in order to identify and compare optimal strategies across a diversity of potential objectives. Constants were varied in increments of 1% of the respective species ‘moderate’ population sizes, proportions were varied in 1% increments, and thresholds also in 1% increments ranging from the respective species ‘quasi-extinction’ population threshold, to a ‘moderate’ population size (see Table S1.2). … Thus, the 18 environmental contexts were each subject to 4 harvest strategies, totalling 72 environment × harvest strategy cases. Overall, we simulated 289, 848 different environment × harvest strategy × harvest parameter models, each with 1000 iterations.” [line 176]

[Comment E12]

- In Figure 4, top three panels, is the composite score comparison for the complete set?

[Reply E12] No – it is across all the composite metric sets, as stated in the figure legend: “Differences in composite score outcomes (x-axis) due to differences in environmental and evaluation factors (y-axis), with all other factors held at equivalent levels for each pairwise contrast” [line 298]. The comparisons of the Complete composite set could be seen through looking at the individual scores in Figure 3.

[Comment E13]

- Line 259, “result in lower composite metric scores, indicating stronger conflicts between objectives”. Can lower composite metric scores occur simply because of low score on all component metrics, i.e., without conflict?

[Reply E13] Yes, thank you for highlighting this omission here. We have modified this to read: “result in lower composite metric scores, indicating either irresolvable poor scores and/or stronger conflicts between objectives” [line 287].

[Comment E14]

- Please refer to Table S1.3 in caption or lead up to Fig. S2.1.1 to define SID1, SID2, etc…

[Reply E14] We have modified the supplementary S2 to include: “Six environmental scenarios were defined for each of the three species. Scenario ID (SID) codes can be found in Figure 1, and fully described in Table S1.3.” [S2 line 6] and also include reference to Table 1 and 2 for descriptions of the individual and composite metrics.

[Comment E15]

- The 6 SID plots within Fig S2.1.1 look the exact same, despite representing different environmental contexts. This is not expected given the sensitivity of performance to environmental context in Fig. 3. The same for Fig. S2.1.2. The figures are not consistent with the text, “Optimum harvest parameters (that maximize the composite metric score) varied across environmental and evaluation contexts (Supplementary S2).” (lines 280-1).

[Reply E15] Thank you for alerting us to a bug in the visualization code, this and the resulting figures have now been corrected.

[Comment E16]

- Fig S.2.2.1 and S2.2.3 Caption/text need to be added. Currently state “Description to be added.. These show the parameter levels that result in top scores (black outline) or within 5% of this top score (coloured areas).” (S2.2.1) and “Description to be added.. These show the parameter levels that result in top scores (black outline) or within 5% of this top score (coloured areas).” (S2.2.3).

[Reply E16] Thank you, this omission has been corrected.

[Comment E17]

- Suggest renumbering Fig S.2.2.3 as S2.2.2 is missing

[Reply E17] Thank you, this has been corrected, we have now separated the constant (S2.2.1), proportional (S2.2.2), and threshold-proportional (S2.2.3) cases for this supplementary.

[Comment E18]

- Line 331 “Pairwise contrasts in environmental factors show that more complex harvest strategies generally become more preferable with faster life history species and higher variability scenarios (Supplementary Fig S2.4).”  This is not obvious from Fig. S2.4. Moving from Moose to Ptarmigan, it looks like there is a high frequency of shift to simpler optimal  harvest rules. Moving from low to high variability it looks like optimal harvest rules generally stay the same in complexity. Figure S2.4 needs a caption and description.

[Reply E18] Thank you for pointing this out. We have now corrected this issue. All pairwise comparison figures (Fig 4,5, S2.3, S2.4) now all have the same format (Factor1 vs. Factor2). This figure now correctly shows Moose systems having more preference to simpler harvest strategies compared with equivalent roe deer systems.

[Comment E19]

- Line 339. Can you clarify what “cases” means in this sentence (all scenarios within 6 harvest rules, optimal harvest parameters, 6 SIDs, 3 species?), “This would be the correct optimal choice in 55.6% of cases, and result in an expected value forgone of 1.19% (Fig 6-7). Proportional, constant, and no harvest strategies would result in a mean value forgone of 2.75%, 12.2%, and 27.0% respectively”

[Reply E19] We have clarified this wording throughout the results. For example, in discussing the optimal harvest strategy, we clarify this to “Overall, the most optimal harvest strategy was threshold proportional, which was optimal in 55.6% of the 108 species × environment × evaluation cases (and intermediate otherwise; Fig 6, comparing across panel columns).” [line 358] In the section referenced above, we echo this wording “This would be the correct optimal choice in 55.6% of the 108 species × environment × evaluation cases, and result in an expected value forgone of 1.19% (Fig 6-7).” [line 371]

- Line 354, Please clarify that these results aggregate over evaluation contexts and species with optimal harvest parameters “If all environmental context information was considered, optimal decisions could be made in 63.9% of cases, with an expected value forgone of 0.57%.”

We have clarified this to “If all environmental context information was considered (i.e. decisions made based on species and environmental context, but not evaluation context)…” [line 386]

# Reviewer #1

[Comment R1.1] I have reviewed the manuscript entitled “sustainability of wildlife harvest in stochastic social-ecological systems” prepared by Law et al. The authors use a MSE framework to simulate the effects of harvest on wildlife under different combinations of environmental, harvest and evaluation contexts. In general, the paper is well written and presented with a lot of simulation work supporting the results. However, I do have a few concerns that need to be addressed before considering for publication in PLOS ONE.

1. The title needs to be more informative. The current title is fairly broad and vague and it’s difficult for the readers to capture what the authors are trying to address in this paper. Furthermore, I don’t think the simulation work done in this paper is adequate to address this broad topic.

[Reply R1.1] We have revised the title to “Heuristics for the sustainable harvest of wildlife in stochastic social-ecological systems”, to highlight our emphasis on developing heuristics.

[Comment R1.2] 2. The purpose of this paper is not quite clear to me. The authors used MSE to simulate and compare outcomes under different combinations of environmental, harvest and evaluation contexts, and then basically conclude the outcome is context-dependent (isn’t this something self-evident?). There are numerous combinations in the simulation and I don’t quite get what is the focus/main question. It is important to have a hypothesis-driven topic in scientific papers, and I encourage the authors to further think about this and reorganize the paper on some specific question/hypothesis/prediction.

[Reply R1.2] We have modified the title to further emphasise our main aim is to develop heuristics for wildlife management in stochastic socio-ecological systems. The abstract clearly states this aim, and why heuristics are useful. We have modified the last paragraph of the introduction to more specifically relay this aim: “To develop heuristics for sustainable terrestrial wildlife harvest, we constructed a semi-complex MSE framework that allowed us to assess sustainability under a range of environmental contexts and from diverse socio-ecological perspectives, and thereby identify patterns of context dependencies over environmental and evaluation contexts… This MSE framework bridges a gap between simplified harvest models with a narrow focus on harvest off-take and highly context-specific applied case studies, with the intention of producing heuristics that are directly applicable to real-world settings” [line 98]

While context-dependency was certainly a likely outcome, our results contribute to the literature by detailing which types of contexts have the most influence. In a decision context, knowledge that outcomes are likely context dependent is not valuable – rather, knowledge on how these contexts are likely to systematically vary is useful. In contrast to prior emphasis in the harvest literature on environmental contexts, we found a greater influence of evaluation contexts on perceived outcomes, optimal harvest parameters, and optimal harvest strategies. We also highlight cases in which adaptive harvest strategies are not the preferred option, in contrast to the majority of simulation cases and existing recommendations from the literature.

We further put this in a decision context, by calculating the likely value forgone by different harvest choices.

[Comment R1.3] 3. For the environmental context (others may refer to operating model in MSE), the authors used logistic model for the population dynamics with 18 combinations of 3 species, 2 levels of variability and 3 starting population sizes. The variability levels apply to 4 parameters (r, m, q and h), so there should be 2^4=16 scenarios of variability, unless the authors assume the 4 parameters are inter-correlated (if so, why?).

[Reply 1.3] One of the reasons why we aimed to develop heuristics is to help guide sensitivity analyses simulation studies to those factors that are likely to make the most difference, as there are a great number of parameters going into them, and thus an arguably impossible range of scenarios to develop and assess if all these are to be systematically varied. Indeed, as we have ‘environmental’ variability in the r,m,q, and h parameters in terms of both stochasticity and parameter uncertainty, total possible variability is 2^8 = 256, many combinations of which are unreasonable (e.g. low r and high h). Couple that with the 6 ‘composite’ evaluation metrics, 4 harvest systems, and many harvest parameters, we feel this is unreasonable to present within one paper.

We therefore chose to only model select variability scenarios, that are indeed inter-correlated. We choose these to give ‘bounds’ to the influence of variability. We added clarification in the methods that we only look at the two variability scenarios: “For each species we simulated two variability scenarios, where variability in *r,* *m, q*, and *h* was either all *low* or all *high*, to represent systems with different extremes of variability and parameter uncertainty.” [line 159]

[Comment R1.4] 4. To introduce stochasticity in the simulation, the authors assume normal distribution of the 4 variable parameters, which is likely to generate negative values. To avoid negative values of abundance et al., a method “round to nearest positive value” is used, but this will generate bias in the simulation. A better practice is to use log-normal distribution to introduce stochasticity.

[Reply R1.4] Normal distribution of errors (stochasticity) are used in our study because we assume these to be a result of a number of factors, which would tend towards a normal distribution (via the central limit theorem). We also selected this because it is a reasonable general assumption, and we intend for future analyses in similar frameworks to investigate the influence of alternative assumptions. One of the issues we have with using lognormal distributions in this case is that we wanted a distribution that could retain the same parameters across multiple values (close to zero, far from zero). Lognormal distributions – while they naturally do not go below zero, are right skewed, which would introduce their own bias. This is certainly a trade-off. In mathematical models, lognormal is typically preferred because it is mathematically more tractable, but in this simulation study, we argue that a truncated normal is indeed a feasible distribution. However, as collectively this source variability was not the main driver of results, we anticipate this choice to have little influence in the overall messages of the manuscript.

We have corrected “round to the nearest positive value” to read “round to the nearest non-negative integer” [S1 e.g. line 15] to indicate the inclusion of zero in this set. This is applied to the results from the logistic growth equation (N_i,t_), the final (adjusted) quota (Q’_i,t_), and the amount harvested (H_i,t_). All of these values are constrained to non-negative integers in reality, and are therefore naturally biased, and we anticipate our (stochastic) model is not unrealistic in this regard.

[Comment R1.5] 5. For the evaluation context, the authors need to further explain how these individual and composite metrics are related to definitions of “sustainability”. If sustainability is the theme of this paper, the authors should try to give their own definition upfront, so that they can better quantify this term in the evaluation context.

[Reply R1.5] We have modified the introduction to more clearly define our definition of sustainability: “In contrast to most previous harvest system models that focus on a narrow set of objectives, we evaluate sustainability over 10 evaluation metrics reflecting volumes and variability of both population abundance and harvest, combined into 6 stakeholder perspectives relevant for terrestrial contexts. In this way, our working definition of sustainability operates at the system level and aims to capture both outcomes and potential conflicts between stakeholders that may moderate these.” [line 104]

[Comment R1.6] 6. When calculating composite metrics, do you give equal weight to individual metrics? If so, how do you balance the number of individual metrics related to population or harvest?

[Reply R1.6] Yes – equal weight is given to all individual metrics included in each composite metric. We chose this approach, as different ‘balance/weighting’ between the objectives is given by the different composite metrics (in that they include or exclude certain individual objectives). This is explained in the methods section, in Table 2, and in detail in supplementary S1. We agree these weightings are arbitrary, but felt for a baseline study equal weighting was appropriate (noting the challenges of weighting in SDM are not insignificant).

[Comment R1.7] 7. In the result section, different composite metric score reflects different perspective on sustainability, and I’m not sure how this can be summarized across composite metrics. In line 234, what is the meaning of 85% of composite metric score? Does this reflect different things among composite metric scores. Line 236, “better performing context” is a weird statement, so some environment/evaluation contexts are better than others?

[Reply R1.7] If we are developing heuristics that are to be applicable over a range of different scenarios (including different evaluation perspectives), then yes, we believe it is appropriate to summarise across composite metrics. If decisions are being made ignoring stakeholder perspectives, then this is a reasonable expectation if there is no further information.

As individual metrics are scaled from 0 to 100% of the maximum possible value, and composite metrics are an average of the included individual metrics, 85% represents a scenario that performs well across the range of included metrics. We clarify this in text as: “Overall, only 11% of the 432 environmental and evaluation context combinations had composite metric scores of over 85% (i.e. performing well across all included metrics), highlighting that poor performances are common, and conflicts between individual metrics, and thus between stakeholder interests, are likely in terrestrial harvest management”. [line 254]

We have changed “better performing context” to “Contexts resulting in high composite scores”. [line 258] In this, we aim to convey that some contexts have more capacity to achieve well on all counts simultaneously (i.e. our definition of sustainability).

[Comment R1.8] 8. The resolution of figure 1 is a bit low.

[Reply R1.8] This has been resolved.

# Reviewer #2:

[Comment R2.1] I had the chance to review the paper “Sustainability of wildlife harvest in stochastic social-ecological systems” for consideration as a Research Article in PLOS ONE. Overall, I really like this paper and recommend that it is suitable for publication with some minor revisions. Great job! The focus of this paper is on using a robust combination social, management, and ecological scenarios of a harvested ecosystem to provide general rules of thumb for decision-making in complex systems. I found the model competently executed, the scenarios explored were interesting, and the paper is very well written. I don’t have many major comments or critiques to the paper. The main comments that I have are on interpreting the visuals and the Discussion text and begin in no particular order below:

[Reply R2.1] Thank you for your support!

[Comment R2.2] Did the authors consider some sort of minimax (MinMax) decision rule to evaluate which harvest strategies minimized the maximum possible loss among all scenarios considered? In this case, the loss function could be the composite score or the average value forgone. I think this would provide a very generalizable heuristic on the decision rules that help to achieve an acceptable level of value across the uncertain range of management and ecological contexts. Is this already captured in Figure 7 with the ‘optimal strategy’?

[Reply R2.2] We agree there are a number of alternative decision strategies that could be undertaken, and would be interesting in this context. For this reason, we included the concept of the value forgone (by choosing one harvest strategy over another), which forms one of the modes of evaluation of the ‘optimal’ strategy, as illustrated in Figure 7. This is a minimization of loss decision strategy, however does not take account of the uncertainty fully, as a formal minimax decision strategy should. We would have liked to include more, however felt the manuscript was already complex enough in it’s current form.

We did also consider a function for the composite metrics of maximizing the minimum outcome – albeit in an earlier version of the manuscript. However we found this additional decision rule resulted in a more complex manuscript (particularly as the minimum outcome in many cases was zero, resulting in many perceptively equivalent scores), and chose to remove it from this version.

[Comment R2.3] What is meant by L. 75 deriving general inferences from case studies? Do you mean that we lack heuristics from empirical case studies because these case studies aren’t experimental? What about meta-analyses?

[Reply R2.3] We have changed this to ‘heuristics’, i.e. “it is challenging to robustly derive heuristics from empirical case studies in wildlife harvest” [line 81], and further clarify this as “because of the conceptual, logistical, and ethical difficulty in conducting systematic experimentation at the scales required (i.e. while it is theoretically possible, there are practical challenges in collating enough comparable studies for comparisons without confounding factors complicating interpretation)” [line 82]. Deriving heuristics requires a systematic understanding of the outcomes from a broad range of scenarios. In complex contexts, such as terrestrial wildlife management, we typically simply do not have the range of empirical experiments to support the development of heuristics – differences between contexts are often confounded. While meta-analyses are valuable in summarizing data, they still rely on having enough (comparable) data to summarise. We now also include this by stating the problems stated above persist “even with meta-analyses” [line 82].

[Comment R2.4] Table 2: in the composite metric set, are the individual metrics weighted equally or unequally? I think L. 200 suggests they are equal. In some fisheries literature (Carruthers et al. 2018; van Poorten & Camp 2019) there is consideration that some metrics should be weighed quite differently (e.g., Persistence weighed 2x more than Harvest Mean). Or, in other words, management may only really care about Persistence when it is low (near some conservation-relevant levels) but not when its high, otherwise we care more about the Harvest Mean (or other criteria).

Carruthers, T. R., Dabrowska, K., Haider, W., Parkinson, E. A., Varkey, D. A., Ward, H., ... & Post, J. R. (2019). Landscape-scale social and ecological outcomes of dynamic angler and fish behaviours: processes, data, and patterns. Canadian Journal of Fisheries and Aquatic Sciences, 76(6), 970-988.

van Poorten, B. T., & Camp, E. V. (2019). Addressing challenges common to modern recreational fisheries with a buffet-style landscape management approach. Reviews in Fisheries Science & Aquaculture, 27(4), 393-416.

[Reply R2.4] We have introduced this topic briefly into the discussion: “This emphasises the importance of identification and weighting of evaluation metrics [(Carruthers et al 2019, van Poorten & Camp 2019)]” [line 447]. We agree that the weighting of individual metrics is certainly complex – more complex than the simple equal weighting we give them here. Our study was designed as a ‘baseline’ and so we deliberately chose ‘simple’ parameters and model forms in this first instance. As the evaluation contexts were very influential in determining what might be perceived as optimal harvest strategies, we indeed suggest exploration into impacts of weighting should be a focus of further study – both in simulated models such as in this current manuscript, and empirically in terms of collation and analysis of real-world weighting in applied cases.

[Comment R2.5] I really like the description of the model methods and procedure. Nicely done.

[Reply R2.6] Thank you! It is nice to hear our efforts in trying to make this as clear as possible have been worthwhile.

[Comment R2.7] I found several of the Figures difficult to make intuitive sense of, particularly Figures 3, 4, 5, and 6. As these are the main results Figures, I suggest either some changes to the figures, the figure captions, or the accompanying text in the main Results to help the reader make these more intuitive to take inferences from. Figures 3 and Figure 6 are quite similar but showcase different metrics (composite scores versus value forgone) so my comments pertain to both.

For Figure 3/6: I am unsure on how the ‘rank’ symbols are meant to be inferred and how the reader is supposed to compare and contrast rows vs. columns vs. colours vs. symbols. Let’s take Figure 6 for example: for Moose managed under Threshold Proportional with a Population Focus: there are 3 ‘clear bests’ outcomes across the environmental contexts and within an environmental context (for example Context 2) there are 2 clear bests and 2 joint bests strategies. What is the main takeaways from this figure that you are trying to communicate to the reader? I am not sure if it is clear how the reader is supposed to read and compare the scenarios. It looks really great though!

[Reply R2.7] Figure 3 presents the maximum composite metric scores possible for all the composite metrics, harvest strategies and environmental scenarios. We agree this is complex, but felt it was useful to have these comparisons for the reader to examine. The symbols here are meant to facilitate interpretation by clearly demarcating the low, medium, and high scores – particularly for colorblind readers, for which such scaled colors can be challenging. In this case, because both the scores and score class can be compared directly across all the cells. In order to make this more interpretable, we add further description to the figure legend: “Composite scores (colour) for each environmental (x-axis, and panel rows) and evaluation context (y-axis), under each harvest strategy (panel column), assuming harvest parameters are optimised under each harvest strategy. Environmental contexts (SID) codes are provided in Fig 1. Score classes (symbols) highlight where scores are maximal (i.e. 100, achieving the highest theoretically possible scores across all included metrics). This figure shows how scores are typically higher for slower life history species (e.g. Moose) than faster life history species (e.g. Ptarmigan), and for more complex harvest strategies (e.g. threshold proportional) than simpler strategies (e.g. constant harvest), but also that few maximal scores were found across the simulated contexts.” [line 273]

Figure 6 is more complex, as symbols and colours do not align. As with the composite metric score in Figure 3, value forgone can similarly be compared across all cells. However, the strategy rank is the relative performance of the different harvest strategies, across a particular species, environmental context, and composite metric. We have amended the figure legend to include further description on interpretation “Strategies are compared for optimality only within equivalent species x environmental x evaluation comparisons. This figure shows how proportional and threshold proportional strategies are typically the most optimal, and typically result in lower value forgone when not.” [line 347]

These two figures are effectively moderately processed results from which we draw our heuristics, and we include them to be transparent regarding the outcomes of all of our contexts. We appreciate these are difficult to draw generalizations from, which is why we follow figure 3 with these summarized comparisons in figures 4 and 5, and in text for Figure 6. We have clarified this in the legends for both figure 3 and 6, with: “Differences are further summarized in text and in the following figures.” [Lines 280, 349]

[Comment R2.8] For Figure 4: I think it shows the difference between composite scores between two scenarios (all else equal). But does a negative difference mean that the left-hand side had the higher score or the lower score? Example of some of my confusion can be illustrated in the first comparison Moose v. Roe Deer: across all scenarios, all else equal, a Moose life history had a negative difference in the composite score than a Roe Deer life history – so does that mean that Moose tended to have the higher score or the lower score? What does the 0.76 and 0.13 mean? And can you walk through what the takeaway here is for Moose, as an example? If I read this right and Moose have a negative difference in the composite score, is the Moose life history harder to manage (under the assumptions of this model) than a Roe Deer life history? I think based on Figure 3 that Moose are slightly easier to manage (likely because they are less variable), so I think part of my challenge can be helped by having the Figure caption or results text better walk through these figures.

[Reply R2.8] As we explain in the legend of figure 4 (and by extension figure 5): “Differences in composite score outcomes (x-axis) due to differences in environmental and evaluation factors (y-axis), with all other factors held at equivalent levels for each pairwise contrast. Contrasts are given change in outcome for the left-hand level vs. the right-hand level, for example, moose typically result in a higher composite metric score than roe deer, all other factors equivalent*.”* [line 297]

The 0.76 and 0.13 are explained also in the legend: “Proportions of the observations below or above zero difference are given on the left and right grey panels respectively (and may not sum to one if some cases do not differ).” [line 303]

[Comment R2.9] For Figure 5: the figure caption discusses optimum harvest parameters. But is this meant to be a sensitivity test? Or what is it? How can you have a difference in the threshold/harvest constant when I think you have set the threshold as an input parameter? And what do the colors represent (there’s no color legend provided). When I see this figure and the axis labels, I though it was showing a sensitivity test but the boxplot seems to indicate it is the outcome of many simulations.

[Reply 2.9] Given decisions on harvesting include both a strategy (e.g. constant, or proportional) and the harvest parameter (e.g. a proportion of 3% or 5% per annum), we wanted to give an indication of how the optimal harvest parameters (i.e. those that give the highest scores) change with respect to the environmental or evaluation contexts, whether they always change in the same direction, and how substantial is the change for each factor. We have amended the figure legend to highlight this is what is shown here: “Thumbnail figure (full figures given in the Supplementary S2.3) showing pairwise differences in optimal harvest parameters (i.e. those parameters giving the highest composite metric scores in each respective context)”. [line 323] We also point towards Figure 4 for the color legend.

These thumbnails in figure 5 are given to indicate that the evaluation factors have a much stronger influence on changing the harvest parameters in a threshold-proportional system, and have relatively little effect (compared with the species and starting population size) on the parameter selected in a constant harvest system. We have amended the figure legend to highlight this: “This overview figure shows how harvest parameter decisions are influenced relatively more by evaluation contexts in more complex harvest strategies (e.g. threshold proportional), compared with a simple ‘constant harvest’ strategy.” [line 329]

[Comment 2.10] Some of the Discussion paragraphs read more like Results. For example, L. 392 mostly focuses on the paper results. I suggest broadening the Discussion out a bit more to engage with the ongoing conversations about adaptive management, monitoring, and decision-making from wildlife and fisheries papers.

[Reply 2.10] We have endeavored to further our discussion’s links to the relevant literature, including via bringing forward the caveats into the main text, and incorporating suggested references, as discussed in the replies above.
