# Supplementary material for: Heuristics for the sustainable harvest of wildlife in stochastic social-ecological systems
Source: PLoS One. 2021 Nov 19;16(11):e0260159. doi: 10.1371/journal.pone.0260159 (PMC8604319; doi:10.1371/journal.pone.0260159)
Supplement: Attachment — Submitted filename: Comments reply 20211013.docx [file pone.0260159.s004.docx]

Response to reviewers

**PONE-D-21-14753R1
Heuristics for the sustainable harvest of wildlife in stochastic social-ecological systems**

[Comments, numbered, aligned, in blue]

[Responses, inset, in green, with text from manuscript in underline]

# Editorial comments

# [Comment E1] Line 191-2. I suggest adding some additional text on these lines “While we aimed to include as broad a range of sustainability metrics as possible, these may not be adequately representative of ecological or socio-economic objectives that are not rooted in volumes or variability of either harvest or population sizes. For example, objectives related to ecosystem function or economic equity may not be captured here” (or something similar).

[Reply E1] We have revised this to read “While we aimed to include as broad a range of sustainability metrics as possible, these may not be adequately representative of ecological or socio-economic objectives that are not a function of volumes or variability of either harvest or population sizes, for example spatial, sex, and age distribution of individuals and consequent effects on ecological functions and equity of opportunity” [line 191].

# [Comment E2] Fig 3 Would you consider putting the SID scenarios in order from low to high. I was confused in my earlier comments, as I assumed SID 3-4 represented moderate initial population sizes, being in the middle (not low). The current order is not intuitive.

[Reply E2] To help resolve this misunderstanding, we have revised the relevant paragraph in the main methods to emphasise the logic of our ordering: “For each species, variability and starting population scenario combinations are identified numerically (SID 1-6) defined in Fig 1. We simulated two variability scenarios, where variability in r, m, q, and h was either all low (SID 1,3,5) or all high (SID 2,4,6), to represent systems with different extremes of variability and parameter uncertainty. Each of these species-variability scenarios were coupled with three distinct scenarios for the population size at the start of the simulation period: 1) moderate (best case), i.e. the midpoint of low and high critical thresholds (SID 1,2), 2) quasi-extinction (SID 3,4), and 3) overabundance (SID 5,6).” [Line 159]

We agree that there are potentially many ways to order the different levels of the variable parameters in this analysis. We selected this order to put the baseline (or our expected ‘easiest’) scenario first (starting at optimal population sizes, with low variation, SID 1). From there, we looked at the different sub-optimal starting population sizes, of ‘too low’ and ‘too high’. To both emphasise that the moderate starting size is the baseline, and to maintain consistency with other material based on this same schema, we wish to retain this order.

# [Comment E3] Fig 4 caption. “Differences in composite score outcomes (x-axis) due to differences in environmental and evaluation factors (y-axis), with all other factors held at equivalent levels for each pairwise contrast”. Apologies, but this caption is still not clear to me. I suggest adding “across all composite metric sets” to clarify (as you mentioned in your reply to E12).

[Reply E3] We have revised this to: “Differences in composite score outcomes (x-axis) due to differences in environmental and evaluation factors (y-axis), with all other factors held at equivalent levels for each pairwise contrast. Contrasts are given as change in the left-hand level vs. right-hand level, for example, moose typically result in a higher composite metric score than roe deer, all other factors equivalent. Violins show the data distributions *(i.e. the values of pairwise contrasts, across all environmental and evaluation factors such as composite metric sets, that are not the focus of the pairwise contrast),* with the colour indicating the median.” [Line 304]

# [Comment E4] Fig S2.1.1 and S2.1.2 Some text in the Supp. Mat. explaining Figures for SID 3 and 4 might helpful, e.g., describing how lines are often not visible because…

[Reply E4] We have added this explanation to the introduction section to Supplementary 2: “As the constant harvest levels differ due to population sizes of the various species, we have scaled these with respect to their respective ‘moderate’ population size. Simulations for constant harvests iterated over increasing constant harvest parameters until all the replicate iterations resulted in population sizes of zero at the end of the time period (i.e. probability of persistence equal to zero; see methods detail). As such, their values do not necessarily span the whole width of the facet window. This is particularly evident under small starting population sizes (SID 3-4) where the small population size effectively limits the possibility of harvesting without driving the population to extinction.” [Supplementary 2 line 8]

# [Comment E5] Fig S2.4. I suggest adding an explanation of the legend. In particular, “+ Simpler”, “-More Complex”, “- Simper”, and “+More Complex” are not intuitive.

[Reply E5] We have revised the legend for Fig S2.4 to include this description: “Harvest strategies are ranked from simple to complex as follows: no harvest, constant harvest, proportional harvest, threshold proportional harvest. Change categories are denoted as “simpler” when the optimal strategy or strategies all become simpler, e.g. when changing from proportional to constant, and “more complex” when the strategies become more complex. As there are often multiple optimal strategies in any particular case, change categories of “+ simpler” or “ – simpler” denote when a simpler harvest strategy is (respectively) added or removed from the optimal strategy set for a particular case. Likewise, “+ more complex” and “ – more complex” represent when a more complex strategy is added or removed from a set.”

# [Comment E6] Discussion or S1 (Section on Caveats). I suggest adding future research that includes other distributions of random variables. I agree with Reviewer 1, that the normal distribution is not the most biologically appropriate approach, but arguably can be used here, at least provisionally, for this study on heuristics.

[Reply E6] We have revised the discussion to include “Further developments of the model would be valuable to extend the results to more complex and realistic contexts, in particular to examine impacts of different distributions and particular sources of uncertainty and variability …” [line 507].
